# Empirical Evaluation of Alternative Time-Series Models for COVID-19 Forecasting in Saudi Arabia

**DOI:** 10.3390/ijerph18168660

**Published:** 2021-08-16

**Authors:** Isra Al-Turaiki, Fahad Almutlaq, Hend Alrasheed, Norah Alballa

**Affiliations:** 1 Department of Information Technology, College of Computer and Information Sciences, King Saud University, Riyadh 11451, Saudi Arabia; halrasheed@ksu.edu.sa; 2 Department of Computer Science, College of Computer and Information Sciences, King Saud University, Riyadh 11451, Saudi Arabia; nalballa@ksu.edu.sa; 3 Geography Department, College of Arts, King Saud University, Riyadh 11451, Saudi Arabia; falmutlaq@ksu.edu.sa

**Keywords:** COVID-19, time-series analysis, autoregressive integrated moving average, TBATS, exponential smoothing, cubic spline, simple exponential smoothing, Holt, HoltWinters

## Abstract

COVID-19 is a disease-causing coronavirus strain that emerged in December 2019 that led to an ongoing global pandemic. The ability to anticipate the pandemic’s path is critical. This is important in order to determine how to combat and track its spread. COVID-19 data is an example of time-series data where several methods can be applied for forecasting. Although various time-series forecasting models are available, it is difficult to draw broad theoretical conclusions regarding their relative merits. This paper presents an empirical evaluation of several time-series models for forecasting COVID-19 cases, recoveries, and deaths in Saudi Arabia. In particular, seven forecasting models were trained using autoregressive integrated moving average, TBATS, exponential smoothing, cubic spline, simple exponential smoothing Holt, and HoltWinters. The models were built using publicly available daily data of COVID-19 during the period of 24 March 2020 to 5 April 2021 reported in Saudi Arabia. The experimental results indicate that the ARIMA model had a smaller prediction error in forecasting confirmed cases, which is consistent with results reported in the literature, while cubic spline showed better predictions for recoveries and deaths. As more data become available, a fluctuation in the forecasting-accuracy metrics was observed, possibly due to abrupt changes in the data.

## 1. Introduction

The recent COVID-19 pandemic was first identified in Wuhan, China, in December 2019, and started to spread globally [1], sparking a series of responses, including countrywide lockdowns, curfews, and travel bans. Although the most common symptoms of COVID-19 infection are mild, it may have serious and even fatal effects on some patients. COVID-19 is a global crisis, with globally more than 179,686,071 confirmed cases and more than 3,899,172 deaths as of 25 June 2021 [2]. The rising number of COVID-19 cases has globally overburdened healthcare facilities, but the virus continues to be poorly understood. Researchers from different fields have been researching the COVID-19 virus since its first appearance.

The lack of historical data that can guide scientists on assessing the disease’s impact and forecasting its future dynamics is a major issue. Predicting the progress of COVID-19 is crucial for public-health planning and decision making. One way to achieve this is by accurately estimating the number of active cases at any given point in time.

Confirmed daily COVID-19 cases, recoveries, and deaths are examples of time-series data. Time-series data consist of a sequence of numeric data, measured at equivalent time periods (e.g., per minute, hour, or day). Many natural and economic processes, such as stock markets and scientific, medical, or natural findings yield time-series outcomes [3].

Statistical and machine-learning time-series models are powerful tools for estimating the progress of diseases, as they can use collected incidence data to predict future occurrences of a disease [4]. They were used to predict the future dynamics of malaria [5,6], influenza [7,8,9], tuberculosis [10,11], and other infectious diseases [12,13]. Recently, time-series models were used to forecast the dynamics of COVID-19 in the USA [14], Italy [14], India [15,16], and several other countries [17,18].

In this work, we use several time-series models for the future forecasting of infection cases, recoveries, and deaths in Saudi Arabia at the country and city levels. The models were applied to publicly available data of daily infections, recoveries, and deaths of COVID-19 from 24 March 2020 to 5 April 2021. For both analytical levels (country and city), we conducted 28-day-ahead point forecasts and fit statistics. We updated our forecasts every four weeks under each of the seven considered models. This eventually created three 28-day forecasting periods.

It is very difficult to draw general theoretical conclusions about the relative merits of various time-series models for COVID-19 forecasting. Thus, the main contribution of this work is to present an empirical evaluation of various time-series models for COVID-19 forecasting in Saudi Arabia and the city of Riyadh (the capital city of Saudi Arabia). Seven well-known time-series models were utilized: autoregressive integrated moving average (ARIMA), exponential smoothing state space model with Box–Cox transformation, ARMA errors, trend and seasonal components (TBATS), exponential smoothing (ETS), cubic spline, simple exponential smoothing (SES) Holt, and HoltWinters. This empirical evaluation was needed in order to suggest a suitable model that could be conveniently used as a public-health guide for COVID-19 prevention and control.

Our results showed good performance of the ARIMA model in predicting the number of confirmed cases, and the cubic-spline model in predicting the numbers of recoveries and deaths. Contrary to what was suggested regarding accuracy increase as more data became available, our results showed that a fluctuation in forecasting-accuracy metrics was observed, possibly due to abrupt changes in the data [14].

The rest of the paper is organized as follows: Section 2 presents related work in using time-series analysis for disease forecasting. In Section 3, we discuss the time-series methodology, which includes the collection and description of the used dataset and models. The performance evaluation of the developed models is discussed in Section 4. Lastly, research findings and implications are discussed in Section 5.

## 2. Literature Review

There are numerous publications in the literature on the use of time-series models to predict pandemics. The ARIMA model is widely used for the short-term predictions of infectious-disease dynamics [4,9,15,17,19], and the SARIMA model is used when temporal trends of seasonality exist in the data [9].

In [9], time-series analysis was used to construct ARIMA and SARIMA models on the basis of monthly influenza incidence from 2004 to 2011 in four provinces in mainland China. The goal was to predict influenza incidences in 2012.

Recently, several research efforts have proposed different time-series models to estimate the spread of COVID-19. For example, in [15], ARIMA was developed to predict the incidences of COVID-19 in India and countries with the highest numbers of confirmed cases, including USA, Spain, Italy, France, Germany, China, and Iran. Analysis was based on daily COVID-19 data that were collected for the period from 22 January 2020 to 13 April 2020. The ARIMA model was more capable in the prediction of COVID-19 cases compared to other prediction models, including instance support vector machine (SVM) and wavelet neural network (WNN). Existing India COVID-19 data were also used for forecasting new daily confirmed cases using two models, earlyR and ARIMA [16]. A comparison between the two models showed that the ARIMA model provided better accuracy than that of the earlyR model.

Four time-series models (Holt, ARIMA, TBATS, and the cubic smoothing spline model) were applied to publicly available daily COVID-19 data for both the USA and Italy [14]. Generally, the authors observed that all models reasonably predicted the future numbers of confirmed cases, deaths, and recoveries. However, the ARIMA and cubic smoothing spline models both had smaller prediction errors for most analyses.

In [20], time-series models based on the two-piece scale mixture normal (TP–SMN) distributions were considered. Historical COVID-19 data were first used to fit the model. Then, the best-fit models were selected and applied to forecast the number of globally confirmed COVID-19 cases. The selected models outperformed the ordinary Gaussian time-series model.

A Bayesian time-series framework to predict the number of COVID-19 infection cases in the USA was proposed [21]. The authors used historical USA data and data from other different countries as prior reference, taking into account the difference in population sizes.

The authors in [18] used Facebook’s Prophet model and the ARIMA model on the number of confirmed cases, deaths, and recoveries to forecast the trend of the disease in Indonesia. When the two models were compared, the results suggested that the Prophet model generally outperformed the ARIMA model, despite it being further from the actual data the more days it forecast. To forecast the dynamics of COVID-19 in Pakistan, a pragmatic approach of the Kalman filter was used with the ARIMA model [17]. This was to avoid the use of assumptions and parameters from which the other approaches suffer.

The prediction of COVID-19 progress in Saudi Arabia is a subject of interest. In [22], an ARIMA model was used to predict the daily number of new COVID-19 infections in Saudi Arabia for the four following weeks. First, a comparison between four different models (autoregressive model (AR), moving average (MA), a combination of both (ARMA), and ARIMA) was performed to determine the best model fit. The authors found that the ARIMA model outperformed the others in predicting the daily number of cases in Saudi Arabia.

In [23], the ARIMA model was used to predict the prevalence of COVID-19 cases in Saudi Arabia using numbers of daily confirmed diagnoses, recoveries, and deaths from 2 March 2020 to 30 June 2020 as reported by the Saudi Ministry of Health. Past and forecast data showed high correlation. The ARIMA and logistic-growth models showed excellent performance in forecasting the prevalence and dynamics of COVID-19 [24]. Using COVID-19 data for the period between 2 March 2020 and 21 June 2020, the authors developed two different scenarios. The first covered the period between 2 March 2020 and 28 May 2020, when the first peak had been observed. The second scenario covered the period between the last week of May and 21 June 2020, when a sudden sharp spike had been observed in the number of new confirmed cases. In another study, the peak of COVID-19 progression in Saudi Arabia was predicted using the Susceptible, Infected, and Recovered (SIR) model [25]. Based on data collected between 2 March and 29 April 2020, COVID-19 in Saudi Arabia was expected to reach a second peak and end around the middle of the year, according to the experimental results. Based on data collected between 2 March and 25 April 2020, a network-based epidemic model for the spread of COVID-19 in Saudi Arabia was made [26]. Many factors were considered in building the model, such as individuals’ social behaviors and dynamics. The study concluded that closing schools and mosques had the most significant impact on delaying the epidemic peak and reducing infection rates. If no vaccine is available by 10 June 2020, and no social distancing is practiced, the predictions suggest the epidemic will end in Saudi Arabia by early November, with over 13 million infected, and it may take only 15 days if 70% of the population receives a vaccine. ARIMA model and Spatial Time-Autoregressive Integrated Moving Average(STARIMA) were utilized to estimate the impact of the curfew on the prevalence of COVID-19 in KSA [27]. The two models were built using confirmed cases from 31 May to 11 October 2020, in the cities of Makkah, Jeddah, and Taif. Experimental results suggested that STARIMA models had superior forecasting ability with regard to future epidemics of COVID-19.

Deep learning models are increasingly used to handle time-series data. Omran et al. [28] used two deep learning methods for predicting COVID-19 confirmed cases and deaths in Egypt, Saudi Arabia, and Kuwait. Time series data collected between 1 May to 6 December 2020 were used to train long short-term memory (LSTM) and gated recurrent unit (GRU).The results show that LSTM performed best in confirmed cases in all three countries, while GRU performed best in death cases in Egypt and Kuwait.

The COVID-19 pandemic in Saudi Arabia was analyzed using modified singular spectrum analysis (SSA) [29]. The study used COVID-19 data from 2 March to 12 May 2020. Results showed a peak around the end of May or June 2020, and the pandemic ending between the end of June and mid-August 2020, with approximately 330,000 infected.

Table 1 summarizes the time-series models reviewed in this paper for predicting COVID-19 infections. While several studies were conducted to forecast COVID-19 spread in Saudi Arabia using time series models, the majority of studies used small datasets. There are numerous time series forecasting models available; however, choosing an appropriate model is not simple. This work fills that gap by examining the utility of several models for forecasting COVID-19 cases in Saudi Arabia using a larger dataset. By comparing multiple models empirically in terms of forecasting accuracy, we hope to recommend a suitable model that can be used to forecast the outbreak’s near future. We recognize that this is a difficult forecasting problem, given the ongoing nature of the pandemic and the fact that there are numerous variables beyond our control at the moment.

## 3. Materials and Methods

Time-series forecasting focuses on analyzing past observations of a random variable to develop a model that captures underlying trends and patterns present in the data. The developed model can then be used to predict future values of the random variable. This type of analysis is very useful when the underlying data-generation process is unknown. In this work, we use several time-series forecasting models to predict future trajectories of COVID-19 in Saudi Arabia.

### 3.1. Data Description

**Figure 1:**
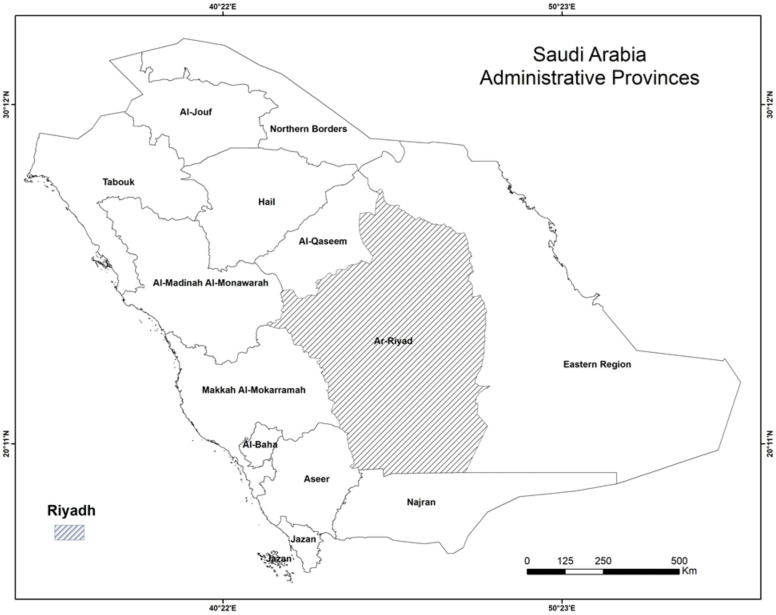
Thirteen administrative provinces in Saudi Arabia with capital city Riyadh shaded in gray.

Saudi Arabia is divided into 13 administrative provinces with a population of 35 million people (Figure 1). The first case of COVID-19 in Saudi Arabia was recorded in March 2020 by the Ministry of Health. This study depends on daily COVID-19 data retrieved from the Saudi Arabian ministry of health COVID-19 response bulletin [30], which provides several sources of data about the COVID-19 pandemic in Saudi Arabia. It includes various sources of information for use in research. The study period of the data used in this paper extends from 24 March 2020 to 5 April 2021 (378 days). The obtained data in dBASE format are then converted into a spreadsheet format. This study focuses on confirmed and recovered COVID-19 cases and deaths for Saudi Arabia (country level), and confirmed cases for Riyadh (city level). This study relied on cumulative daily data for confirmed, recovered, and death cases of COVID-19 (see Table 2 as an example). Riyadh is the capital of Saudi Arabia that has a population of 8 million individuals, so the most COVID-19 confirmed cases were recorded within it.

### 3.2. Time-Series Analysis Models

The ordered sequence of a variable’s values that are gathered at equally spaced time periods is referred to as a time series. Time-series analysis entails building models that characterize the observed time series in order to obtain a better understanding of the underlying factors. Time-series analysis serves several purposes. Its core functions include: (1) finding patterns or characteristics that lead to phenomena, (2) anticipating changes in the series, and (3) controlling the pattern or feature that resulted in the phenomenon. To help us forecast the number of COVID-19 infections, we provide a brief description of several time-series models.

#### 3.2.1. Simple Exponential Smoothing (SES)

Simple exponential smoothing [31] is a method for univariate time-series forecasting where trend or seasonality is not observed in the data. Weighted averages are used to predict future values on the basis of the most recent ones, with those values given greater weight. Observations made in the past are given less weighting. SES is calculated as follows:

(1) $$F_{t}=F_{t-1}+\alpha \left(A_{t-1}-F_{t-1}\right)$$

where $A_{t}$ is the actual value of the series at time *t*, $F_{t}$ is the forecast value of the series at time *t*, and α is a weighting parameter that takes a value between 0 and 1. A larger value of α indicates that recent-series values are given more weight. SES is appropriate for a series that moves randomly above and below a constant mean. It has no trend and no seasonal patterns [31,32].

#### 3.2.2. Autoregressive Integrated Moving Average (ARIMA)

The ARIMA model is a frequently used time-series forecasting model that was proposed in 1970 [33]. An ARIMA model is a generic linear stochastic model that combines autoregressive, moving-average models, and differencing in order to achieve stationary time series [34]. A typical autoregressive model takes previous values and uses a linear combination of those values to forecast the future values of the variable of interest. The moving-average model employs the previous forecasts’ errors in a manner similar to that of a regression model [35]. ARIMA provides realistic results when the data show no seasonality [9,22]. The general notation for ARIAM is ARIMA(p,d,q), where *p* is used to calculate AR using *p* preceding periods from the time series, *d* represents the degree of differencing that is used to transform the data into a stationary series, and *q* is the order of the moving average. Forecasting using ARIMA is calculated as follows [35]:

(2) $$y_{t}=c+\varphi_{1}y_{t-1}+\ldots +\varphi_{p}y_{t-p}+\theta_{1}e_{t-1}+\ldots +\theta_{q}e_{t-q}+e_{t}$$

where $y_{t}$ is the difference at degree *d* of the original series of time series, $\varphi_{1}-\varphi_{p}$ are autoregressive model parameters, $\theta_{1}-\theta_{q}$ represent moving-average model parameters, and $e_{t}$ is white noise.

#### 3.2.3. Exponential Smoothing State Space Model with Box–Cox Transformation, ARMA Errors, Trend and Seasonal Components (TBATS)

ARIMA and exponential smoothing, which are the most used models, are only capable of handling one seasonality. Varying seasonal trends are often seen in time series (e.g., hourly data that contain a daily, weekly, and annual pattern). TBATS is a time-series method that is capable of modeling complex and multiseasonal time-series data. “TBATS” is the abbreviation for the models’ salient characteristics: trigonometric seasonality, Box–Cox transformation, ARMA errors, and trend and seasonal components [36]. TBATS uses exponential smoothing to forecast data with complex seasonal patterns.

#### 3.2.4. Exponential Smoothing (ETS)

Exponential smoothing [37,38,39], is a typical statistical approach for the prediction of time-series data. The ETS technique is particularly beneficial for datasets that contain seasonality and other data-related assumptions. ETS predicts using a weighted average of all observations in the input time-series dataset. Weights exponentially drop over time, as opposed to fixed weights used in basic moving-average approaches. Weights are determined by a constant value called the smoothing parameter.

#### 3.2.5. Cubic Spline

Cubic spline is based on a stochastic state-space model that allows for the estimation of the smoothing parameter using a likelihood technique. The cubic-spline model can be considered to be a special case of an ARIMA(0, 2, 2) model [40]. It offers better smoothing of long-term trends and linearity for predictions. Given a univariate time series $y_{t}$, for t=1,…,n, the cubic spline is a function that minimizes

(3) $$\sum_{t=1}^{n}{\left(y_{t}-f\left(t\right)\right)}^{2}+\lambda \int_{S}{\left(f^{{}^{\prime \prime }}\left(u\right)\right)}^{2}du$$

over all twice differentiable functions *f* on *S* where [1,n]⊆S⊆R. λ regulates the exchange rate between the residual error characterized by the sum of squared residuals and local variation, which is measured by the square of the second derivative of *f*.

#### 3.2.6. Holt and HoltWinters

Holt’s linear trend [41] is a generalization of the basic exponential-smoothing approach that enables trend-based forecasting. It is the application of exponential smoothing to both the series’ average value (level) and trend. It includes three equations that work together to generate a forecast. The first is $l_{t}$, the level equation, which is a basic smoothing equation, calculated as follows:

(4) $$l_{t}=\alpha y_{t}+\left(1-\alpha \right)\left(l_{t-1}+b_{t-1}\right)$$

The second is $b_{t}$, the trend equation used to update the trend and it is calculated as follows:

(5) $$b_{t}=\beta^{*}\left(l_{t}-l_{t-1}\right)+\left(1-\beta^{*}\right)b_{t-1}$$

where α and $\beta^{*}$ are smoothing parameters for the level and trend, respectively, falling in the range of 0–1, inclusive. Lastly, the third equation is used to generate the final forecast for the horizon h as follows [42]:

(6) $$F_{t+h|t}=l_{t}+hb_{t}$$

The HoltWinters model is an extension of the Holt model that adds the seasonality factor to the forecast.

### 3.3. Experimental Settings

All models discussed here were implemented using the forecast package in R. The code used for our analyses is provided in the Supplementary File 1. All experiments were run using a MacBook Pro with the macOS Catalina operating system, version 10.15.7, and a 2.9 GHz quad-core Intel Core i7 with 16 GB RAM.

### 3.4. Performance Measures

The forecasting performance of each model was evaluated using the three following measures:

- Root mean square error (RMSE)—the square root of the mean of the square of all the errors.
  (7) $$RMSE=\sqrt{\frac{1}{n}\sum_{t=1}^{n}{\left(A_{t}-F_{t}\right)}^{2}}$$
  where $A_{t}$ and $F_{t}$ are the actual and forecasted values of the series at time *t*, respectively.
- Mean absolute error (MAE)—the average of absolute errors in a dataset. It is calculated as follows:
  (8) $$MAE=\frac{1}{n}\sum_{t=1}^{n}\left|A_{t}-F_{t}\right|$$
- Mean absolute percentage error (MAPE)—the accuracy of the forecasting model as a ratio, calculated as follows:
  (9) $$MAPE=\frac{1}{n}\sum_{t=1}^{n}\left|\frac{A_{t}-F_{t}}{A_{t}}\right|$$

## 4. Results

### 4.1. Experiment Results

We conducted forecasting analysis for confirmed COVID-19 cases, recoveries, and deaths for Saudi Arabia (country level), and confirmed cases for Riyadh (city level). The models were trained using a training dataset of 294 days, from 24 March 2020 to 11 January 2021 to perform 28-day-ahead forecasts. Our forecasts were updated every four weeks under each of the seven considered models, which eventually created three 28-day forecasting periods from 12 January 2021 to 5 April 2021. In addition, The figures for all models are provided in the Supplementary File 2, for better visualization.

#### 4.1.1. Confirmed Cases in Saudi Arabia

**Figure 2:**
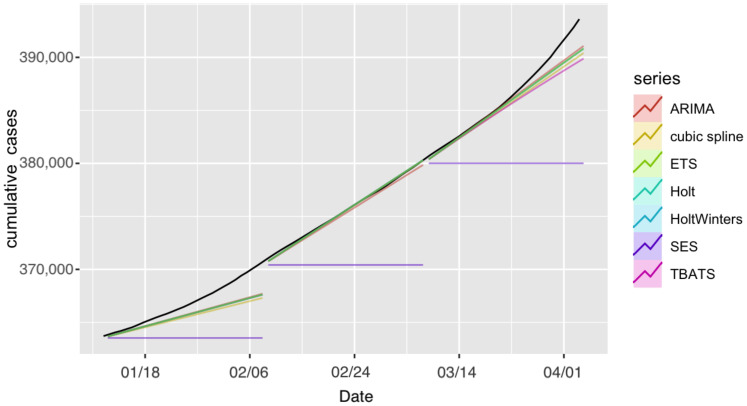
Forecast confirmed cases in Saudi Arabia projected under each model and forecasting period. The black line represents observed confirmed cases.

**Figure 3:**
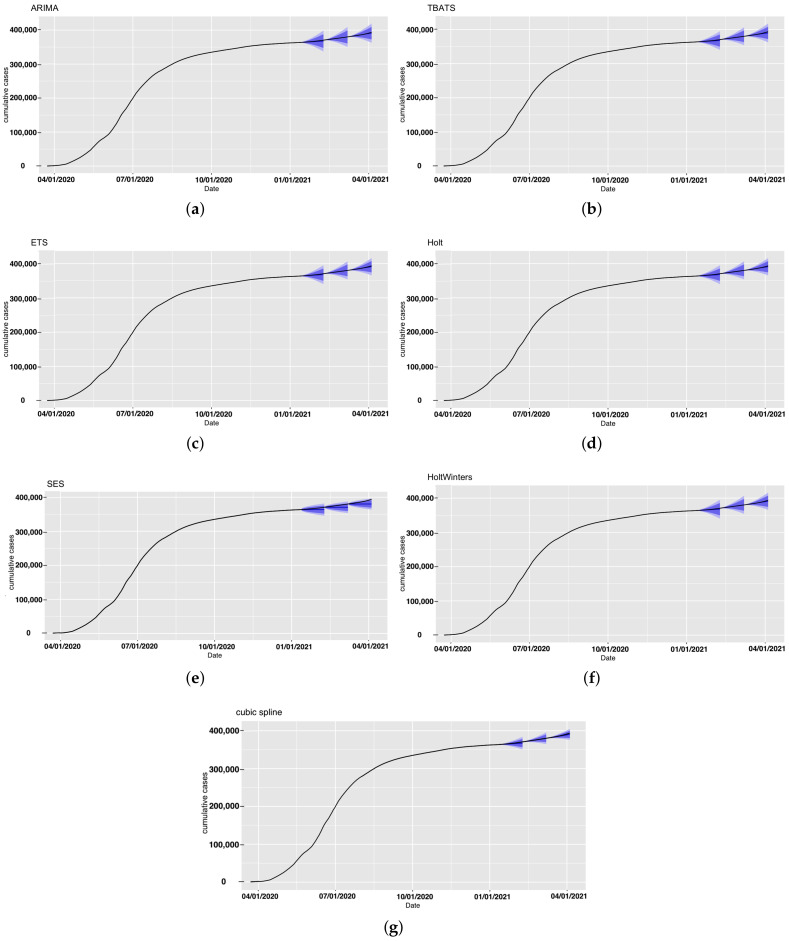
Forecast confirmed cases in Saudi Arabia for all models with the prediction intervals (in blue). (**a**) ARIMA, (**b**) TBATS, (**c**) ETS, (**d**) Holt, (**e**) SES, (**f**) HoltWinters, and (**g**) cubic spline.

First, we employed the seven models to forecast the number of confirmed cases for Saudi Arabia. The observed and forecast confirmed COVID-19 cases in Saudi Arabia are presented in Figure 2. The forecasts for each model with prediction intervals (PIs) are presented in Figure 3. The forecasting-accuracy metrics for this application are summarized in Table 3. Overall, the most accurate estimation was obtained using ARIMA for the second forecast period, covering from 9 February to 8 March 2021.

Now, we look at each period individually. The first forecast period covered 12 January–8 February 2021. The ARIMA model achieved the most accurate estimation for this specific forecasting period and had the smallest prediction errors, as shown in Table 3. In second place were ETS, Holt, and HoltWinters, which achieved similar performance.

In the second forecast period, covering 9 February–8 March 2021, we incorporated four more weeks of historical data and included the number of cases observed until 8 February 2021 to perform 28-day ahead predictions. Figure 2 and Figure 3 show the produced forecasts at the end of 8 February. Similar to the previous period, ARIMA achieved the most accurate estimation and the best performance in the testing set, with a MAPE value of 0.01% and RMSE value of 54.67, and MAE value of 41.8. Except for the SES model, all other models had good performance in this period, and the estimations were close to the actual number of confirmed cases.

For the third forecast period, we considered forecasting for 9 March–5 April 2021, by using the data up until 8 March 2021. The ARIMA model achieved the best performance, with a MAPE value of 0.138% and RMSE value of 865.6, and had the most accurate estimation compared to the actual number of cases.

All algorithms except SES produced better predictions during the second period. This could have been due to the sudden unexpected rise in the number of confirmed cases in the other periods. For all three forecasting periods, all prediction models exhibited consistent performance with the exception of the SES. The SES model had poor performance compared to the six other models in the analyses. SES models are only useful for nonseasonal patterns with no trend and for short-term forecasting, since any prediction beyond the next period must utilize the predicted value for that period as a proxy for the actual demand. As a result, no correction information can be added, and any mistake exponentially increases. The forecasts for the seven models for the confirmed cases for the three 28-day forecasting periods and the actual number of cases are provided in Tables S1 and S2 in the Supplementary File 1.

#### 4.1.2. Recoveries in Saudi Arabia

**Figure 4:**
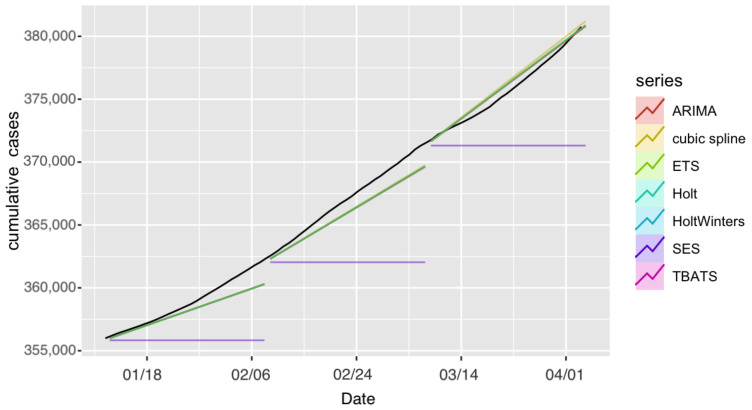
Forecast recoveries in Saudi Arabia projected under each model and forecasting period. Black line represents observed recoveries.

We next applied the same models to generate forecasts for the number of recoveries for COVID-19 in Saudi Arabia. The prediction performance of all models is summarized in Table 4, and the observed and forecast recoveries in Saudi Arabia are presented in Figure 4. The forecasts for the seven models for the recovered cases for the three 28-day forecasting periods and the actual number of cases are provided in Tables S1 and S3 in Supplementary File 1.

**Figure 5:**
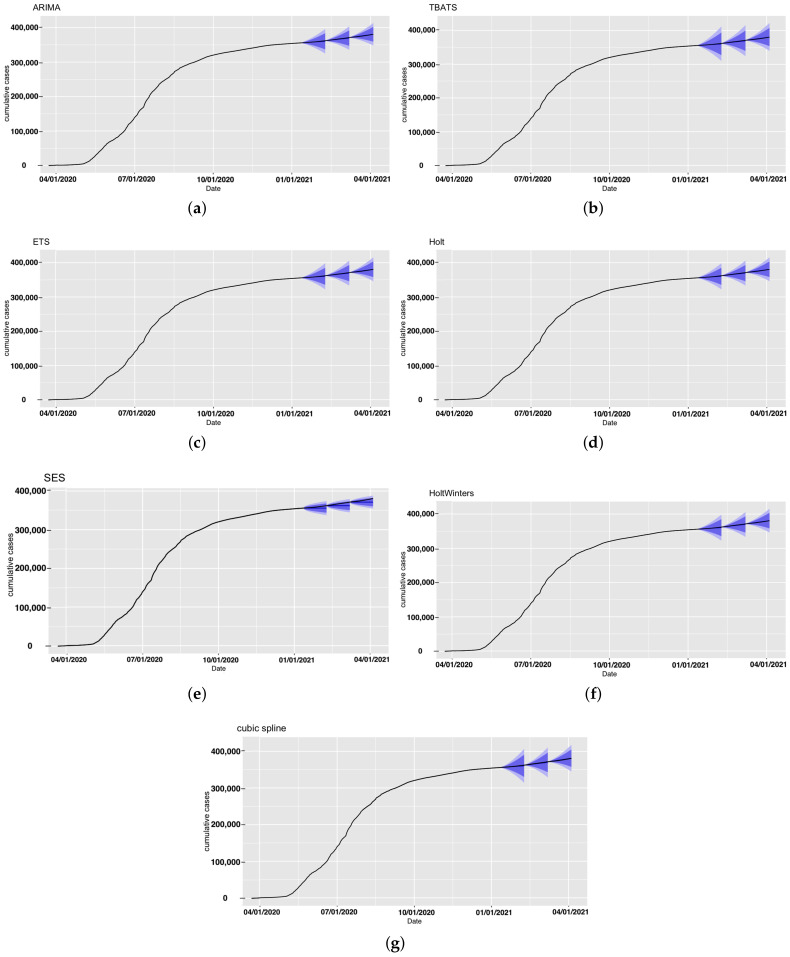
Forecast recoveries in Saudi Arabia for all models with the prediction intervals (in blue). (**a**) ARIMA, (**b**) TBATS, (**c**) ETS, (**d**) Holt, (**e**) SES, (**f**) HoltWinters, and (**g**) cubic spline.

Table 4 shows that all models, with the exception of the SES model, achieved similar performance. The values of the actual observed number of recoveries were within the PIs of all seven models for all three forecasting periods (Figure 5). The prediction performance of the ARIMA and cubic-spline models was slightly better than the performance of the other models.

Table 4 also shows a decrease in forecast accuracy in Period 2 compared to that in Period 1, and an increase in accuracy in Period 3 compared to that in Period 2. Generally, all models achieved their best prediction performance in Period 3.

#### 4.1.3. Deaths in Saudi Arabia

**Figure 6:**
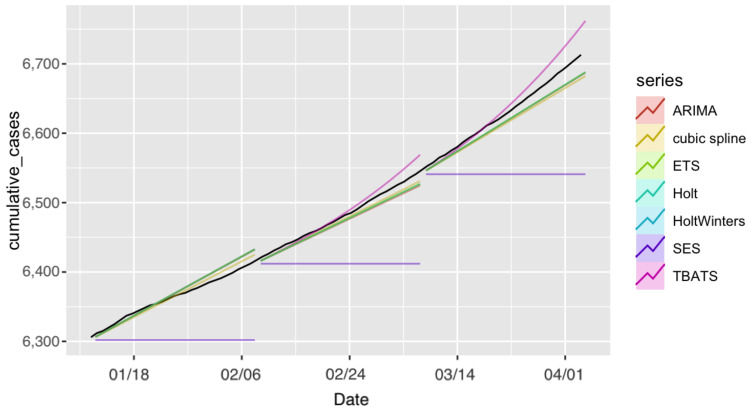
Forecast deaths in Saudi Arabia projected under each model and forecasting period. Black line represents the observed deaths.

We conducted forecasting for the number of deaths due to COVID-19 in Saudi Arabia using the same set of models. Forecasting-accuracy metrics for this application are summarized in Table 5, and the forecast and the actual deaths in Saudi Arabia are presented in Figure 6. The actual number of death cases and forecasts for all models are provided in Tables S1 and S4, respectively, in Supplementary File.

**Figure 7:**
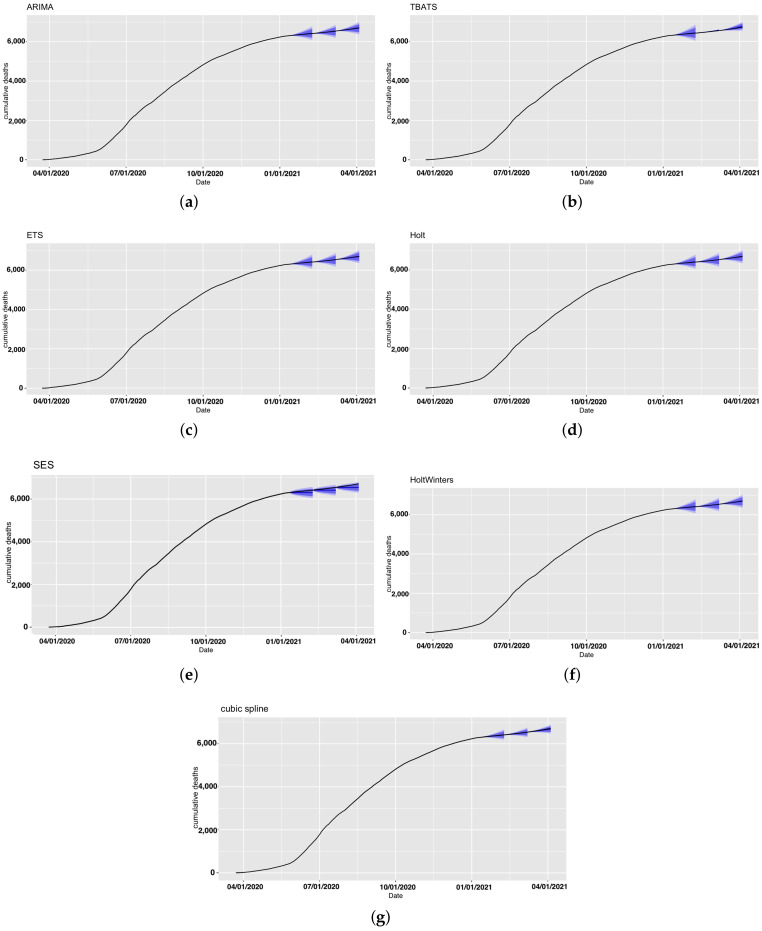
Forecast deaths in Saudi Arabia for all models with prediction intervals (in blue). (**a**) ARIMA, (**b**) TBATS, (**c**) ETS, (**d**) Holt, (**e**) SES, (**f**) HoltWinters, and (**g**) cubic spline.

The prediction performance of the cubic-spline model was slightly better than that of the other models in the first two periods. For the last period, the ETS, Holt, HoltWinters, and ARIMA models achieved better performance than that of the other models. Again, the SES model yielded the worst accuracy. However, the PIs of all models included the observed number of deaths for all periods (Figure 7).

Similar to forecasting confirmed cases in Saudi Arabia, the best performance of all the models was achieved in Period 2. An increase in forecast accuracy in Period 2 compared to that in Period 1 was observed. Moreover, a decrease in forecast accuracy in Period 3 compared to that in Period 2 was observed.

#### 4.1.4. Confirmed Cases in Riyadh

**Figure 8:**
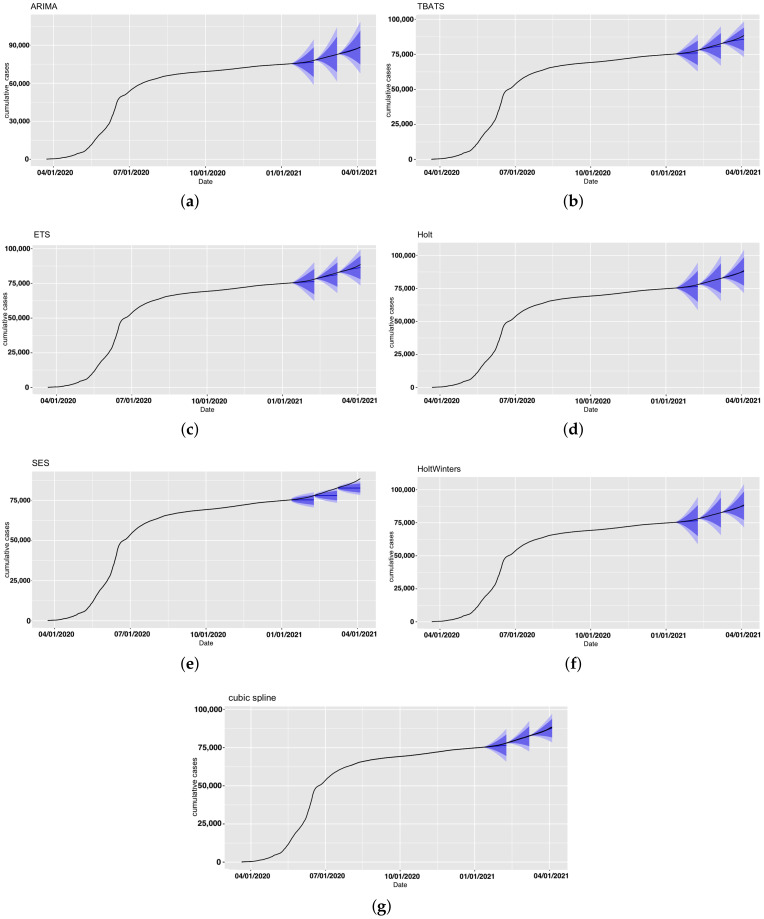
Forecast confirmed cases in Riyadh for all models with prediction intervals (in blue). (**a**) ARIMA, (**b**) TBATS, (**c**) ETS, (**d**) Holt, (**e**) SES, (**f**) HoltWinters, and (**g**) cubic spline.

**Figure 9:**
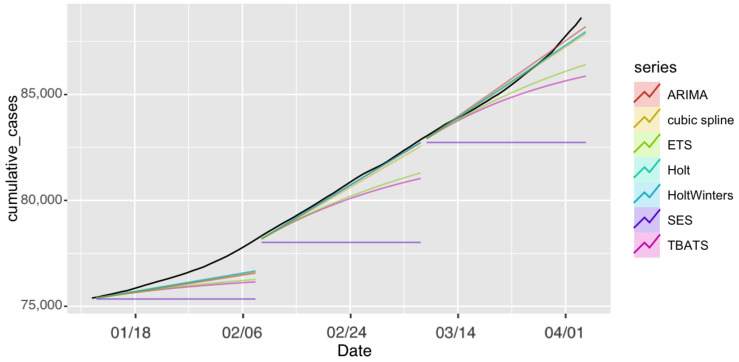
Forecast confirmed cases in Riyadh projected under each model and forecasting period. Black line represents observed confirmed cases.

We performed forecasting analysis for confirmed COVID-19 cases in Riyadh using the same models and forecasting periods. Results are presented in Figure 8 and Figure 9.

Overall, for this application, the Holt and HoltWinters, ARIMA, and cubic-spline models achieved good performance compared to the TBATS, ETS, and SES models. The SES model again yielded the highest prediction errors for all three forecasting periods. Looking at overall period results, almost all models performed best in the second period, as the increase rate was consistent with that in the previous period. PIs from all models contained the observed number of confirmed cases for all forecast periods except part of the last period of the SES model (Figure 8). Forecasting-accuracy metrics are summarized in Table 6. The forecasts for the seven models for all forecasting periods and the actual number of cases are provided in Tables S5 and S6 in Supplementary File.

### 4.2. Discussion

Saudi Arabia has already implemented several preventative measures and established a health-surveillance system against COVID-19. In spite of these policies, COVID-19 still prevails with an indefinite transmission pattern. At the time of writing this manuscript, Saudi Arabia (specifically the city of Riyadh) is experiencing an alarming increase in the number of COVID-19 cases. The main purpose of this work is to predict the future dynamics of COVID-19 in Saudi Arabia by applying a set of commonly used statistical-analysis models based on historical disease data. The goal is to understand the trends of this pandemic and assist the authorities in the decision-making process.

We evaluated several time-series models for forecasting confirmed COVID-19 cases, number of deaths, and number of recoveries for Saudi Arabia and Riyadh. Results on the impact of environmental factors, such as seasonal cycle, on the spread of COVID-19 are inconclusive [43]. Therefore, we included time-series forecasting models without trend or seasonality (ARIMA, SES, Cubic splines), with seasonality (TBATS, ETS, HoltWinters), and with trend (Holt). We updated our forecasts every 28 days under each considered model. We eventually created three 28-day forecasting periods from 12 January 2021 to 5 April 2021. The first forecast period covered 12 January–8 February 2021, where we used the data collected during previous days. For the second forecast period, we incorporated 28 more days of historical data by including the number of cases in each category, observed until 8 February 2020. That time, the forecast period covered 9 February–8 March 2021. For the third period, we considered forecasting for 9 March–5 April 2021, and used data up until 8 March 2021.

Generally, all models resulted in similar conclusions; however, each model exhibited subtle differences. The ARIMA model had a smaller prediction error in forecasting confirmed cases. This finding is consistent with results reported in the literature [4,9,15,17,19]. Strong performance could also be observed from the cubic-spline, Holt, and HoltWinters models in the majority of analyses. All models exhibited similar PIs; however, the cubic-spline and SES models had narrower PIs compared to those of the other models. Narrower periods correspond to a higher degree of forecasting certainty. However, the SES model seemed to always yield the worst performance compared to that of the other models. As more data became available from forecasting Periods 1 to 3, a fluctuation in forecasting-accuracy metrics was observed. However, the third period showed higher accuracy compared to that in the first period. This is a result of more data becoming available [14].

There are some limitations in our forecasting approach. First, forecasting presented in this work is short-term (28 days), and it may not be accurate or reliable for long-term forecasting. The models can be enhanced by adding new monitoring data for COVID-19 incidences. Second, pinpointing the exact reasons why certain models perform better than others is not straightforward. Some factors that may play a role in forecasting robustness include climatic and geographical characteristics, population-related attributes such as population density, and implemented preventive measures such as quarantine and other social-distancing measures.

In addition, despite the fact that our analysis employed a relatively large amount of data compared with other studies (e.g., [14,15,23,24,44]), it is still not enough to understand the novel virus and accurately predict its future behavior. Some studies suggested that the virus appears to be temperature-sensitive, and a decline in new COVID-19 cases during summer is expected (e.g., [45,46]). However, this was not the case in Saudi Arabia, as winter months exhibited fewer cases than spring and summer months did. Recent data indicate a significant increase in the number of cases starting from March in both 2020 and 2021 in Saudi Arabia. This may suggest another possible pattern, but it remains unclear due to the limited data and difficulties in separating the impact of social distancing and other preventive measures.

Saudi Arabia launched its most extensive vaccination campaign against COVID-19 in 500 centers with around 15 million doses [47] at the time of writing this script, which should hopefully curb the spread of the virus. Understanding the virus trend and seasonality is essential to track the disease and tailor appropriate measures to contain it.

## 5. Conclusions

COVID-19 has globally spread, posing a major public-health threat. Since the pandemic is ongoing, there is still an urgent need for forecasting models that could help predict more probable pandemic waves. In this paper, we investigated time-series models for forecasting COVID-19 infections by conducting an empirical evaluation. Several models were developed using a dataset from Saudi Arabia. The obtained forecasting results indicate that the ARIMA model had a smaller forecasting prediction error in the majority of analyses.

Unstable patterns in historical data (perhaps due to sudden changes in preventive measures) are more likely to worsen the accuracy of forecasts. However, time-series models that are available to use at any time, for any country, and at multiple scales provide reasonable accuracy forecasts.

Additionally, the cubic-spline, Holt, and HoltWinters models performed well in the majority of our experiments. The findings of this study could be extended by studying other models, such as deep learning. In addition, our models can be updated by incorporating more factors, such as health status, demographics, and environmental factors. It is also worth investigating how time-series models perform across countries.

## Figures and Tables

**Table 1:** Related work using time-series models for infectious-disease forecasting.

Study	Country	Models	Dataset
[15]	India, USA, Spain, Italy, France, Germany, China, Iran, others	ARIMA, SVM, WNN	22 Jan to 13 Apr 2020 (81 days)
[16]	India	earlyR, ARIMA	3–24 May 2020 (21 days)
[14]	USA, Italy	Holt, ARIMA, TBATS, cubic smoothing spline	22 Feb to 29 Apr 2020 (67 days)
[20]	Global	TP–SMN, Gaussian time series	22 Jan to 8 Apr 2020 (77 days)
[21]	Global	Bayesian	21 Jan to 26 Mar 2020 (65 days)
[18]	Indonesia	Facebook’s Prophet, ARIMA	20 Jan to 21 May 2020 (122 days)
[17]	Pakistan	pragmatic approach of the Kalman filter, ARIMA	26 Feb to 30 Apr 2020 (64 days)
[22]	Saudi Arabia	AR, MA, ARMA, ARIMA	2 Mar to 20 Apr 2020 (49 days)
[23]	Saudi Arabia	ARIMA	2 Mar to 30 Jun 2020 (120 days)
[24]	Saudi Arabia	ARIMA and logistic-growth models	2 Mar to 21 Jun 2020 (111 days)
[25]	Saudi Arabia	SIR	2 Mar to 29 Apr 2020 (58 days)
[26]	Saudi Arabia	network-based model	2 Mar to 25 Apr 2020 (54 days)
[27]	Saudi Arabia	ARIMA, STARIMA	31 May to 11 Oct 2020 (58 days)
[28]	Egypt, Saudi Arabia, and Kuwait	LSTM, GRU	1 May to 6 Dec 2020 (219 days)
[29]	Saudi Arabia	SSA	2 Mar to 12 May 2020 (71 days)

**Table 2:** Cumulative numbers at the end of each month in Saudi Arabia and Riyadh for confirmed, recovered, and death cases of COVID-19.

KSA				Riyadh	
Date	Confirmed	Recovered	Deaths	Confirmed	Percentage of the Total Confirmed Cases
31 March 2020	1563	165	10	573	37%
30 April 2020	24,097	3555	169	4524	19%
31 May 2020	85,261	62,474	503	20,927	25%
30 June 2020	190,823	130,766	1649	47,457	25%
31 July 2020	275,905	235,658	2866	54,780	20%
31 August 2020	315,772	290,796	3897	57,391	18%
30 September 2020	334,605	319,154	4768	58,652	18%
31 October 2020	347,282	333,842	5402	59,930	17%
30 November 2020	357,360	346,802	5896	61,722	17%
31 December 2020	362,741	353,853	6223	62,896	17%
31 January 2021	368,074	359,573	6375	64,460	18%
28 February 2021	377,383	368,305	6494	67,634	18%
31 March 2021	390,007	378,083	6669	69,330	18%

**Table 3:** Performance of the proposed models to predict confirmed KSA cases. The best accuracy values are shown in bold.

	Period 1			Period 2			Period 3		
Model	RMSE	MAE	MAPE	RMSE	MAE	MAPE	RMSE	MAE	MAPE
ARIMA	**1225.9**	**921.3**	**0.250**	**54.67**	**41.8**	**0.01**	**865.6**	**539.7**	**0.138**
TBATS	1281.39	972.3	0.264	251.71	216.59	0.057	1397.4	849.1	0.217
ETS	1274.1	965.8	0.262	255.15	219.5	0.058	976.48	588.67	0.151
Cubic splines	1460	1136.44	0.309	223.68	192.73	0.051	1175.3	698.36	0.179
Holt	1274.15	965.83	0.262	255.16	219.5	0.058	976.03	588.46	0.151
HoltWinters	1274.16	965.84	0.262	255.10	219.4	0.058	976.26	588.57	0.151
SES	3672.4	3085.1	0.839	5611.9	4907.9	1.302	7192.1	6070	1.562

**Table 4:** Performance of proposed models to predict KSA recoveries. The best accuracy values are shown in bold.

	Period 1			Period 2			Period 3		
Model	RMSE	MAE	MAPE	RMSE	MAE	MAPE	RMSE	MAE	MAPE
ARIMA	**771.63**	**529.84**	**0.147**	980.55	841.21	0.228	**678.52**	**607.44**	**0.162**
TBATS	779.25	537.41	0.149	960.41	824.15	0.224	697.27	627.98	0.167
ETS	783.04	540.17	0.150	949.71	813.38	0.221	727.31	659.30	0.175
Cubic splines	**769.71**	**526.29**	**0.146**	**898.9**	**766.4**	**0.208**	895.64	829.17	0.220
Holt	783.03	540.17	0.150	949.69	813.35	0.221	727.23	659.22	0.175
HoltWinters	783.01	540.14	0.150	949.72	813.39	0.221	727.23	659.23	0.175
SES	3381.3	2846.7	0.791	5471	4765	1.294	5085.2	4302.9	1.140

**Table 5:** Performance of proposed models to predict KSA deaths. The best accuracy values are shown in bold.

	Period 1			Period 2			Period 3		
Model	RMSE	MAE	MAPE	RMSE	MAE	MAPE	RMSE	MAE	MAPE
ARIMA	11.16	8.36	0.131	7.917	6.5	0.10	**12.13**	**9.6**	**0.144**
TBATS	11.56	8.68	0.136	12.04	8.76	0.134	22.09	16.06	0.241
ETS	11.51	8.64	0.135	6.64	5.31	0.082	**11.64**	**9.19**	**0.138**
Cubic splines	**7.24**	**5.64**	**0.0884**	**4.38**	**3.22**	**0.049**	14.93	12.19	0.183
Holt	11.51	8.64	0.135	6.64	5.31	0.082	**11.64**	**9.19**	**0.138**
HoltWinters	11.51	8.64	0.135	6.64	5.31	0.082	**11.64**	**9.19**	**0.138**
SES	66.66	59.44	0.932	74.58	64.9	0.999	98.56	85.37	1.283

**Table 6:** Performance of proposed models to predict confirmed Riyadh cases. The best accuracy values are shown in bold.

	Period 1			Period 2			Period 3		
Model	RMSE	MAE	MAPE	RMSE	MAE	MAPE	RMSE	MAE	MAPE
ARIMA	645.1	488.4	0.633	**29.17**	**23.89**	**0.029**	300.5	265.9	0.31
TBATS	841.36	633.1	0.821	814.67	626.3	0.768	1105.07	728.89	0.836
ETS	782	586.22	0.760	686.59	528.25	0.648	838.60	518.99	0.594
Cubic splines	616.84	462	0.599	93	79.65	0.98	**262.1**	**199.87**	**0.231**
Holt	**587.59**	**435.72**	**0.565**	**29.57**	**23.89**	**0.0296**	**259.09**	**213.44**	**0.248**
HoltWinters	**587.55**	**435.69**	**0.565**	**29.56**	**23.88**	**0.0296**	**259.1**	**213.6**	**0.248**
SES	1358.3	1120.2	1.455	2780	2428.4	2.991	3102.6	2630.2	3.045

## Data Availability

The dataset used in this study is available at https://github.com/israksu/COVID19Data, accessed on 10 August 2021.

## Supplementary Materials

The following are available online at https://www.mdpi.com/article/10.3390/ijerph18168660/s1, Table S1: KSA actual cases, Table S2: KSA forecasted confirmed cases, Table S3: KSA forecasted recoveries, Table S4: KSA forecasted deaths, Table S5: Riyadh forecasted confirmed cases, and Table S6: Riyadh actual cases.
