# Supplementary material for: Empirical Evaluation of Alternative Time-Series Models for COVID-19 Forecasting in Saudi Arabia
Source: Int J Environ Res Public Health. 2021 Aug 16;18(16):8660. doi: 10.3390/ijerph18168660 (PMC8393561; doi:10.3390/ijerph18168660)
Supplement: Supplementary file 1 [file ijerph-18-08660-s001.zip › Supplementary File 1.pdf]

Supplementary File 1

Table S1: KSA actual cases

	Interval#1			Interval#2			Interval#3		
DAY	Confirmed	Deaths	Recovered	Confirmed	Deaths	Recovered	Confirmed	Deaths	Recovered
1	363709	6306	355989	370794	6417	362344	380396	6547	371559
2	363878	6312	356177	371158	6422	362618	380786	6553	371826
3	364051	6315	356358	371511	6426	362923	381146	6558	372193
4	364191	6320	356517	371848	6431	363279	381497	6565	372432
5	364367	6325	356663	372170	6435	363561	381845	6569	372679
6	364537	6331	356824	372484	6440	363902	382190	6575	372902
7	364763	6337	356980	372806	6443	364273	382544	6580	373106
8	365001	6340	357153	373140	6447	364622	382937	6587	373337
9	365213	6344	357313	373467	6452	364993	383318	6593	373577
10	365426	6348	357501	373804	6456	365339	383709	6598	373840
11	365623	6352	357704	374129	6459	365721	384091	6604	374111
12	365809	6354	357915	374444	6463	366070	384458	6611	374388
13	366022	6357	358113	374771	6468	366388	384862	6615	374775
14	366245	6361	358316	375106	6472	366711	385272	6620	375141
15	366461	6365	358521	375459	6477	366991	385738	6626	375447
16	366714	6368	358729	375815	6482	367299	386220	6632	375807
17	366981	6370	358982	376161	6485	367667	386730	6639	376179
18	367251	6374	359275	376499	6490	367987	387232	6645	376534
19	367512	6377	359549	376821	6496	368281	387763	6652	376923
20	367767	6381	359815	377138	6502	368616	388304	6658	377280
21	368077	6385	360086	377440	6507	368902	388860	6665	377690
22	368383	6388	360376	377771	6512	369253	389445	6671	378059
23	368686	6391	360673	378146	6516	369589	390035	6678	378445
24	369013	6395	360930	378530	6521	369898	390763	6686	378849
25	369399	6399	361213	378912	6526	370276	391447	6692	379288
26	369716	6404	361491	379269	6530	370590	392120	6699	379792
27	370072	6408	361789	379620	6536	371008	392815	6706	380281
28	370425	6412	362038	380010	6541	371314	393607	6713	380748

Table S2: KSA forecasted confirmed cases

	Interval#1						
	ARIMA_1	TBATS_1	ETS_1	cubic spline_1	Holt_1	SES_1	HoltWinters_1
1	363686.1	363679.7	363680.1	363657.5	363680.1	363532.5	363680.0506
2	363836.3	363825.3	363826.1	363792.6	363826.1	363532.5	363826.1011
3	363982.9	363970.9	363972.2	363927.7	363972.2	363532.5	363972.1517
4	364131.2	364116.5	364118.2	364062.7	364118.2	363532.5	364118.2022
5	364281.8	364262.1	364264.3	364197.8	364264.3	363532.5	364264.2528
6	364431	364407.7	364410.3	364332.9	364410.3	363532.5	364410.3033
7	364578.8	364553.3	364556.4	364468	364556.4	363532.5	364556.3539
8	364727.6	364698.9	364702.4	364603.1	364702.4	363532.5	364702.4044
9	364877.4	364844.5	364848.5	364738.2	364848.5	363532.5	364848.455
10	365026.3	364990.1	364994.5	364873.3	364994.5	363532.5	364994.5055
11	365174.6	365135.7	365140.6	365008.3	365140.6	363532.5	365140.5561
12	365323.6	365281.3	365286.6	365143.4	365286.6	363532.5	365286.6066
13	365473	365426.9	365432.7	365278.5	365432.7	363532.5	365432.6572
14	365621.8	365572.4	365578.7	365413.6	365578.7	363532.5	365578.7077
15	365770.5	365718	365724.8	365548.7	365724.8	363532.5	365724.7583
16	365919.5	365863.6	365870.8	365683.8	365870.8	363532.5	365870.8088
17	366068.6	366009.2	366016.9	365818.8	366016.9	363532.5	366016.8594
18	366217.5	366154.8	366162.9	365953.9	366162.9	363532.5	366162.9099
19	366366.3	366300.4	366309	366089	366309	363532.5	366308.9605
20	366515.3	366446	366455	366224.1	366455	363532.5	366455.011
21	366664.3	366591.6	366601.1	366359.2	366601.1	363532.5	366601.0616
22	366813.2	366737.2	366747.1	366494.3	366747.1	363532.5	366747.1121
23	366962.1	366882.8	366893.2	366629.4	366893.2	363532.5	366893.1627
24	367111	367028.4	367039.2	366764.4	367039.2	363532.5	367039.2132
25	367260	367174	367185.3	366899.5	367185.3	363532.5	367185.2638
26	367408.9	367319.6	367331.3	367034.6	367331.3	363532.5	367331.3143
27	367557.8	367465.2	367477.4	367169.7	367477.4	363532.5	367477.3649
28	367706.8	367610.8	367623.4	367304.8	367623.4	363532.5	367623.4154

	Interval#2						
	ARIMA_2	TBATS_2	ETS_2	cubic spline_2	Holt_2	SES_2	HoltWinters_2
1	370762.1	370777.8	370778	370772.4	370778	370421.4	370777.9685
2	371091.5	371130.5	371130.9	371123.6	371130.9	370421.4	371130.9371
3	371429	371483.3	371483.9	371474.8	371483.9	370421.4	371483.9056
4	371771.2	371836	371836.9	371826.1	371836.9	370421.4	371836.8741
5	372107.2	372188.8	372189.9	372177.3	372189.9	370421.4	372189.8427
6	372440.6	372541.6	372542.8	372528.5	372542.8	370421.4	372542.8112
7	372778.5	372894.3	372895.8	372879.8	372895.8	370421.4	372895.7798
8	373117.8	373247.1	373248.8	373231	373248.8	370421.4	373248.7483
9	373453.9	373599.8	373601.7	373582.2	373601.7	370421.4	373601.7168
10	373789.3	373952.6	373954.7	373933.4	373954.7	370421.4	373954.6854
11	374127	374305.4	374307.7	374284.7	374307.7	370421.4	374307.6539
12	374464.9	374658.1	374660.7	374635.9	374660.7	370421.4	374660.6224

13	374801.2	375010.9	375013.6	374987.1	375013.6	370421.4	375013.591
14	375137.5	375363.6	375366.6	375338.4	375366.6	370421.4	375366.5595
15	375475	375716.4	375719.6	375689.6	375719.6	370421.4	375719.528
16	375812.3	376069.2	376072.5	376040.8	376072.6	370421.4	376072.4966
17	376148.9	376421.9	376425.5	376392	376425.5	370421.4	376425.4651
18	376485.6	376774.7	376778.5	376743.3	376778.5	370421.4	376778.4337
19	376822.8	377127.4	377131.5	377094.5	377131.5	370421.4	377131.4022
20	377159.9	377480.2	377484.4	377445.7	377484.4	370421.4	377484.3707
21	377496.6	377833	377837.4	377797	377837.4	370421.4	377837.3393
22	377833.5	378185.7	378190.4	378148.2	378190.4	370421.4	378190.3078
23	378170.6	378538.5	378543.3	378499.4	378543.4	370421.4	378543.2763
24	378507.5	378891.2	378896.3	378850.6	378896.3	370421.4	378896.2449
25	378844.4	379244	379249.3	379201.9	379249.3	370421.4	379249.2134
26	379181.3	379596.8	379602.3	379553.1	379602.3	370421.4	379602.1819
27	379518.3	379949.5	379955.2	379904.3	379955.2	370421.4	379955.1505
28	379855.3	380302.3	380308.2	380255.6	380308.2	370421.4	380308.119

	**Interval#3**						
	ARIMA	TBATS	ETS	cubic spline	Holt	SES	HoltWinters
1	380391.1	380382.9	380396.1	380375.8	380396.2	380006.1	380396.1414
2	380783.2	380753.7	380782.2	380747.4	380782.3	380006.1	380782.2827
3	381189.1	381131.6	381168.4	381119	381168.5	380006.1	381168.4241
4	381586.1	381506.4	381554.5	381490.7	381554.6	380006.1	381554.5654
5	381974.4	381876.6	381940.6	381862.3	381940.8	380006.1	381940.7068
6	382369.6	382246	382326.7	382234	382327	380006.1	382326.8481
7	382770.3	382614.1	382712.9	382605.6	382713.1	380006.1	382712.9895
8	383165.6	382979.5	383099	382977.2	383099.3	380006.1	383099.1308
9	383557.6	383342.9	383485.1	383348.9	383485.4	380006.1	383485.2722
10	383953.7	383704.4	383871.2	383720.5	383871.6	380006.1	383871.4135
11	384351.7	384064	384257.4	384092.1	384257.7	380006.1	384257.5549
12	384746.7	384421.5	384643.5	384463.8	384643.9	380006.1	384643.6962
13	385140.6	384777.1	385029.6	384835.4	385030.1	380006.1	385029.8376
14	385536.8	385130.8	385415.7	385207.1	385416.2	380006.1	385415.9789
15	385933.4	385482.5	385801.9	385578.7	385802.4	380006.1	385802.1203
16	386328.5	385832.2	386188	385950.3	386188.5	380006.1	386188.2616
17	386723.3	386180.1	386574.1	386322	386574.7	380006.1	386574.403
18	387119.3	386526	386960.2	386693.6	386960.9	380006.1	386960.5443
19	387515.3	386870	387346.4	387065.3	387347	380006.1	387346.6857
20	387910.6	387212.1	387732.5	387436.9	387733.2	380006.1	387732.827
21	388305.9	387552.4	388118.6	387808.5	388119.3	380006.1	388118.9684
22	388701.7	387890.8	388504.7	388180.2	388505.5	380006.1	388505.1097
23	389097.4	388227.3	388890.9	388551.8	388891.7	380006.1	388891.2511
24	389492.8	388562	389277	388923.4	389277.8	380006.1	389277.3924
25	389888.3	388894.8	389663.1	389295.1	389664	380006.1	389663.5338
26	390284	389225.8	390049.2	389666.7	390050.1	380006.1	390049.6751
27	390679.6	389555	390435.4	390038.4	390436.3	380006.1	390435.8165
28	391075	389882.4	390821.5	390410	390822.5	380006.1	390821.9578

Table S3: KSA forecasted recoveries

	Interval#1						
	ARIMA_1	TBATS_1	ETS_1	cubic spline_1	Holt_1	SES_1	HoltWinters_1
1	355991.6	355991.3	355992	355997.6	355992	355831.5	355991.985
2	356151.4	356150.4	356151	356157.2	356151	355831.5	356150.9699
3	356311.2	356309.5	356309.9	356316.8	356310	355831.5	356309.9549
4	356471	356468.6	356468.9	356476.5	356468.9	355831.5	356468.9398
5	356630.7	356627.7	356627.9	356636.1	356627.9	355831.5	356627.9248
6	356790.5	356786.9	356786.9	356795.7	356786.9	355831.5	356786.9097
7	356950.3	356946.1	356945.9	356955.3	356945.9	355831.5	356945.8947
8	357110	357105.3	357104.9	357115	357104.9	355831.5	357104.8797
9	357269.8	357264.5	357263.8	357274.6	357263.9	355831.5	357263.8646
10	357429.6	357423.7	357422.8	357434.2	357422.8	355831.5	357422.8496
11	357589.4	357582.9	357581.8	357593.8	357581.8	355831.5	357581.8345
12	357749.1	357742.2	357740.8	357753.5	357740.8	355831.5	357740.8195
13	357908.9	357901.5	357899.8	357913.1	357899.8	355831.5	357899.8044
14	358068.7	358060.8	358058.8	358072.7	358058.8	355831.5	358058.7894
15	358228.4	358220.1	358217.7	358232.4	358217.7	355831.5	358217.7743
16	358388.2	358379.4	358376.7	358392	358376.7	355831.5	358376.7593
17	358548	358538.7	358535.7	358551.6	358535.7	355831.5	358535.7443
18	358707.8	358698.1	358694.7	358711.2	358694.7	355831.5	358694.7292
19	358867.5	358857.5	358853.7	358870.9	358853.7	355831.5	358853.7142
20	359027.3	359016.9	359012.7	359030.5	359012.7	355831.5	359012.6991
21	359187.1	359176.3	359171.6	359190.1	359171.6	355831.5	359171.6841
22	359346.9	359335.7	359330.6	359349.7	359330.6	355831.5	359330.669
23	359506.6	359495.2	359489.6	359509.4	359489.6	355831.5	359489.654
24	359666.4	359654.6	359648.6	359669	359648.6	355831.5	359648.639
25	359826.2	359814.1	359807.6	359828.6	359807.6	355831.5	359807.6239
26	359985.9	359973.6	359966.6	359988.2	359966.6	355831.5	359966.6089
27	360145.7	360133.1	360125.5	360147.9	360125.5	355831.5	360125.5938
28	360305.5	360292.7	360284.5	360307.5	360284.5	355831.5	360284.5788

	Interval#2						
	ARIMA_2	TBATS_2	ETS_2	cubic spline_2	Holt_2	SES_2	HoltWinters_2
1	362305.3	362306.7	362310.4	362322.8	362310.4	362035.5	362310.3547
2	362576	362578.2	362582.7	362597.7	362582.7	362035.5	362582.7094
3	362846.6	362849.7	362855.1	362872.7	362855.1	362035.5	362855.0641
4	363117.3	363121.3	363127.4	363147.6	363127.4	362035.5	363127.4188
5	363388	363392.8	363399.8	363422.5	363399.8	362035.5	363399.7735
6	363658.6	363664.5	363672.1	363697.4	363672.1	362035.5	363672.1282
7	363929.3	363936.1	363944.5	363972.3	363944.5	362035.5	363944.4829
8	364200	364207.9	364216.8	364247.2	364216.9	362035.5	364216.8376
9	364470.6	364479.6	364489.2	364522.1	364489.2	362035.5	364489.1923
10	364741.3	364751.4	364761.6	364797	364761.6	362035.5	364761.547
11	365012	365023.3	365033.9	365071.9	365033.9	362035.5	365033.9017
12	365282.7	365295.1	365306.3	365346.9	365306.3	362035.5	365306.2564

13	365553.3	365567.1	365578.6	365621.8	365578.6	362035.5	365578.6111
14	365824	365839	365851	365896.7	365851	362035.5	365850.9658
15	366094.7	366111	366123.3	366171.6	366123.4	362035.5	366123.3204
16	366365.3	366383.1	366395.7	366446.5	366395.7	362035.5	366395.6751
17	366636	366655.2	366668	366721.4	366668.1	362035.5	366668.0298
18	366906.7	366927.3	366940.4	366996.3	366940.4	362035.5	366940.3845
19	367177.3	367199.5	367212.8	367271.2	367212.8	362035.5	367212.7392
20	367448	367471.7	367485.1	367546.1	367485.1	362035.5	367485.0939
21	367718.7	367744	367757.5	367821.1	367757.5	362035.5	367757.4486
22	367989.4	368016.3	368029.8	368096	368029.9	362035.5	368029.8033
23	368260	368288.6	368302.2	368370.9	368302.2	362035.5	368302.158
24	368530.7	368561	368574.5	368645.8	368574.6	362035.5	368574.5127
25	368801.4	368833.4	368846.9	368920.7	368846.9	362035.5	368846.8674
26	369072	369105.9	369119.2	369195.6	369119.3	362035.5	369119.2221
27	369342.7	369378.4	369391.6	369470.5	369391.6	362035.5	369391.5768
28	369613.4	369650.9	369664	369745.4	369664	362035.5	369663.9315

	**Interval#3**						
	ARIMA	TBATS	ETS	cubic spline	Holt	SES	HoltWinters
1	371647	371648.8	371656	371676.6	371656	371310.9	371656.0059
2	371985.8	371988.6	371998	372029.7	371998	371310.9	371998.0119
3	372324.6	372328.4	372340	372382.8	372340	371310.9	372340.0178
4	372663.5	372668.3	372682	372735.8	372682	371310.9	372682.0238
5	373002.3	373008.3	373024.1	373088.9	373024	371310.9	373024.0297
6	373341.2	373348.2	373366.1	373442	373366	371310.9	373366.0357
7	373680	373688.3	373708.1	373795	373708	371310.9	373708.0416
8	374018.8	374028.4	374050.1	374148.1	374050	371310.9	374050.0476
9	374357.7	374368.5	374392.1	374501.2	374392.1	371310.9	374392.0535
10	374696.5	374708.7	374734.1	374854.2	374734.1	371310.9	374734.0595
11	375035.4	375048.9	375076.1	375207.3	375076.1	371310.9	375076.0654
12	375374.2	375389.2	375418.1	375560.4	375418.1	371310.9	375418.0714
13	375713	375729.6	375760.1	375913.4	375760.1	371310.9	375760.0773
14	376051.9	376070	376102.2	376266.5	376102.1	371310.9	376102.0832
15	376390.7	376410.4	376444.2	376619.6	376444.1	371310.9	376444.0892
16	376729.6	376750.9	376786.2	376972.6	376786.1	371310.9	376786.0951
17	377068.4	377091.4	377128.2	377325.7	377128.1	371310.9	377128.1011
18	377407.2	377432	377470.2	377678.8	377470.1	371310.9	377470.107
19	377746.1	377772.7	377812.2	378031.8	377812.1	371310.9	377812.113
20	378084.9	378113.4	378154.2	378384.9	378154.1	371310.9	378154.1189
21	378423.8	378454.1	378496.2	378738	378496.1	371310.9	378496.1249
22	378762.6	378794.9	378838.2	379091	378838.1	371310.9	378838.1308
23	379101.4	379135.8	379180.3	379444.1	379180.1	371310.9	379180.1368
24	379440.3	379476.7	379522.3	379797.2	379522.1	371310.9	379522.1427
25	379779.1	379817.6	379864.3	380150.2	379864.1	371310.9	379864.1487
26	380118	380158.6	380206.3	380503.3	380206.1	371310.9	380206.1546
27	380456.8	380499.7	380548.3	380856.4	380548.1	371310.9	380548.1605
28	380795.6	380840.8	380890.3	381209.4	380890.2	371310.9	380890.1665

Table S4: KSA forecasted deaths

	Interval#1						
	ARIMA_1	TBATS_1	ETS_1	cubic spline_1	Holt_1	SES_1	HoltWinters_1
1	6306.485	6306.576	6306.568	6306.201	6306.568	6301.95	6306.568324
2	6311.288	6311.251	6311.243	6310.602	6311.243	6301.95	6311.243184
3	6315.803	6315.925	6315.918	6315.003	6315.917	6301.95	6315.918045
4	6320.578	6320.601	6320.593	6319.403	6320.592	6301.95	6320.592906
5	6325.118	6325.276	6325.268	6323.804	6325.267	6301.95	6325.267767
6	6329.871	6329.952	6329.943	6328.204	6329.941	6301.95	6329.942628
7	6334.431	6334.628	6334.618	6332.605	6334.616	6301.95	6334.617489
8	6339.166	6339.305	6339.293	6337.006	6339.291	6301.95	6339.29235
9	6343.743	6343.982	6343.968	6341.406	6343.966	6301.95	6343.96721
10	6348.462	6348.659	6348.643	6345.807	6348.64	6301.95	6348.642071
11	6353.053	6353.337	6353.318	6350.207	6353.315	6301.95	6353.316932
12	6357.76	6358.015	6357.992	6354.608	6357.99	6301.95	6357.991793
13	6362.361	6362.693	6362.667	6359.009	6362.664	6301.95	6362.666654
14	6367.059	6367.372	6367.342	6363.409	6367.339	6301.95	6367.341515
15	6371.669	6372.051	6372.017	6367.81	6372.014	6301.95	6372.016376
16	6376.358	6376.73	6376.692	6372.21	6376.688	6301.95	6376.691236
17	6380.976	6381.41	6381.367	6376.611	6381.363	6301.95	6381.366097
18	6385.659	6386.09	6386.042	6381.012	6386.038	6301.95	6386.040958
19	6390.283	6390.77	6390.717	6385.412	6390.712	6301.95	6390.715819
20	6394.959	6395.451	6395.392	6389.813	6395.387	6301.95	6395.39068
21	6399.588	6400.132	6400.067	6394.213	6400.062	6301.95	6400.065541
22	6404.261	6404.813	6404.742	6398.614	6404.737	6301.95	6404.740402
23	6408.894	6409.495	6409.417	6403.015	6409.411	6301.95	6409.415263
24	6413.562	6414.177	6414.092	6407.415	6414.086	6301.95	6414.090123
25	6418.199	6418.86	6418.767	6411.816	6418.761	6301.95	6418.764984
26	6422.865	6423.542	6423.441	6416.216	6423.435	6301.95	6423.439845
27	6427.504	6428.225	6428.116	6420.617	6428.11	6301.95	6428.114706
28	6432.167	6432.909	6432.791	6425.018	6432.785	6301.95	6432.789567

	Interval#2						
	ARIMA_2	TBATS_2	ETS_2	cubic spline_2	Holt_2	SES_2	HoltWinters_2
1	6415.92	6416.259	6416.169	6416.31	6416.169	6411.96	6416.168846
2	6420.047	6420.507	6420.271	6420.556	6420.271	6411.96	6420.270697
3	6423.986	6424.819	6424.372	6424.802	6424.372	6411.96	6424.372548
4	6428.095	6429.2	6428.474	6429.048	6428.474	6411.96	6428.4744
5	6432.051	6433.655	6432.576	6433.295	6432.576	6411.96	6432.576251
6	6436.146	6438.191	6436.678	6437.541	6436.677	6411.96	6436.678102
7	6440.115	6442.812	6440.78	6441.787	6440.779	6411.96	6440.779954
8	6444.197	6447.524	6444.881	6446.033	6444.881	6411.96	6444.881805
9	6448.177	6452.332	6448.983	6450.279	6448.982	6411.96	6448.983656
10	6452.249	6457.242	6453.085	6454.525	6453.084	6411.96	6453.085507
11	6456.238	6462.26	6457.187	6458.771	6457.186	6411.96	6457.187359
12	6460.303	6467.39	6461.288	6463.017	6461.287	6411.96	6461.28921

13	6464.299	6472.638	6465.39	6467.263	6465.389	6411.96	6465.391061
14	6468.357	6478.009	6469.492	6471.509	6469.491	6411.96	6469.492913
15	6472.359	6483.509	6473.594	6475.755	6473.592	6411.96	6473.594764
16	6476.411	6489.142	6477.695	6480.002	6477.694	6411.96	6477.696615
17	6480.418	6494.914	6481.797	6484.248	6481.796	6411.96	6481.798467
18	6484.466	6500.83	6485.899	6488.494	6485.898	6411.96	6485.900318
19	6488.477	6506.895	6490.001	6492.74	6489.999	6411.96	6490.002169
20	6492.522	6513.114	6494.103	6496.986	6494.101	6411.96	6494.104021
21	6496.536	6519.492	6498.204	6501.232	6498.203	6411.96	6498.205872
22	6500.578	6526.034	6502.306	6505.478	6502.304	6411.96	6502.307723
23	6504.594	6532.745	6506.408	6509.724	6506.406	6411.96	6506.409575
24	6508.634	6539.63	6510.51	6513.97	6510.508	6411.96	6510.511426
25	6512.652	6546.693	6514.611	6518.216	6514.609	6411.96	6514.613277
26	6516.69	6553.939	6518.713	6522.463	6518.711	6411.96	6518.715129
27	6520.71	6561.374	6522.815	6526.709	6522.813	6411.96	6522.81698
28	6524.746	6569.001	6526.917	6530.955	6526.914	6411.96	6526.918831

	Interval#3						
	ARIMA	TBATS	ETS	cubic spline	Holt	SES	HoltWinters
1	6546.37	6546.586	6546.258	6545.859	6546.258	6540.949	6546.257605
2	6551.447	6551.983	6551.508	6550.916	6551.508	6540.949	6551.507476
3	6556.789	6557.829	6556.758	6555.974	6556.757	6540.949	6556.757347
4	6561.891	6563.546	6562.007	6561.031	6562.007	6540.949	6562.007218
5	6567.21	6569.673	6567.257	6566.088	6567.257	6540.949	6567.257089
6	6572.332	6575.726	6572.507	6571.145	6572.507	6540.949	6572.50696
7	6577.633	6582.16	6577.757	6576.203	6577.757	6540.949	6577.756831
8	6582.773	6588.569	6583.007	6581.26	6583.007	6540.949	6583.006702
9	6588.058	6595.334	6588.257	6586.317	6588.257	6540.949	6588.256573
10	6593.211	6602.116	6593.507	6591.374	6593.507	6540.949	6593.506445
11	6598.484	6609.235	6598.757	6596.432	6598.757	6540.949	6598.756316
12	6603.649	6616.408	6604.007	6601.489	6604.006	6540.949	6604.006187
13	6608.911	6623.903	6609.257	6606.546	6609.256	6540.949	6609.256058
14	6614.085	6631.484	6614.507	6611.603	6614.506	6540.949	6614.505929
15	6619.339	6639.376	6619.757	6616.661	6619.756	6540.949	6619.7558
16	6624.521	6647.382	6625.007	6621.718	6625.006	6540.949	6625.005671
17	6629.768	6655.691	6630.257	6626.775	6630.256	6540.949	6630.255542
18	6634.956	6664.138	6635.506	6631.832	6635.506	6540.949	6635.505414
19	6640.197	6672.883	6640.756	6636.89	6640.756	6540.949	6640.755285
20	6645.39	6681.787	6646.006	6641.947	6646.006	6540.949	6646.005156
21	6650.627	6690.985	6651.256	6647.004	6651.256	6540.949	6651.255027
22	6655.824	6700.361	6656.506	6652.061	6656.505	6540.949	6656.504898
23	6661.058	6710.029	6661.756	6657.118	6661.755	6540.949	6661.754769
24	6666.258	6719.892	6667.006	6662.176	6667.005	6540.949	6667.00464
25	6671.488	6730.046	6672.256	6667.233	6672.255	6540.949	6672.254511
26	6676.691	6740.409	6677.506	6672.29	6677.505	6540.949	6677.504382
27	6681.919	6751.063	6682.756	6677.347	6682.755	6540.949	6682.754254
28	6687.125	6761.941	6688.006	6682.405	6688.005	6540.949	6688.004125

Table S5: Riyadh forecasted confirmed cases

	Interval#1						
	ARIMA_1	TBATS_1	ETS_1	cubic spline_1	Holt_1	SES_1	HoltWinters_1
1	75386.26	75390.96	75391.32	75390.37	75392.72	75345.52	75392.72405
2	75427.07	75435.1	75436.14	75435.81	75439.97	75345.52	75439.97509
3	75472.53	75477.6	75479.8	75481.25	75487.22	75345.52	75487.22613
4	75518.9	75518.52	75522.33	75526.7	75534.47	75345.52	75534.47717
5	75562.26	75557.92	75563.75	75572.14	75581.71	75345.52	75581.7282
6	75604.15	75595.86	75604.09	75617.58	75628.96	75345.52	75628.97924
7	75647.74	75632.4	75643.38	75663.02	75676.21	75345.52	75676.23028
8	75692.84	75667.57	75681.65	75708.46	75723.46	75345.52	75723.48132
9	75737.15	75701.45	75718.92	75753.9	75770.71	75345.52	75770.73235
10	75780.2	75734.06	75755.23	75799.35	75817.96	75345.52	75817.98339
11	75823.44	75765.47	75790.59	75844.79	75865.21	75345.52	75865.23443
12	75867.63	75795.71	75825.03	75890.23	75912.46	75345.52	75912.48547
13	75911.94	75824.83	75858.57	75935.67	75959.71	75345.52	75959.7365
14	75955.62	75852.86	75891.24	75981.11	76006.95	75345.52	76006.98754
15	75999.04	75879.86	75923.06	76026.55	76054.2	75345.52	76054.23858
16	76042.83	75905.86	75954.06	76072	76101.45	75345.52	76101.48962
17	76086.91	75930.89	75984.25	76117.44	76148.7	75345.52	76148.74065
18	76130.8	75954.99	76013.65	76162.88	76195.95	75345.52	76195.99169
19	76174.45	75978.2	76042.29	76208.32	76243.2	75345.52	76243.24273
20	76218.15	76000.55	76070.18	76253.76	76290.45	75345.52	76290.49377
21	76262.04	76022.07	76097.35	76299.21	76337.7	75345.52	76337.7448
22	76305.95	76042.79	76123.81	76344.65	76384.95	75345.52	76384.99584
23	76349.73	76062.74	76149.58	76390.09	76432.19	75345.52	76432.24688
24	76393.46	76081.95	76174.68	76435.53	76479.44	75345.52	76479.49792
25	76437.27	76100.45	76199.13	76480.97	76526.69	75345.52	76526.74895
26	76481.13	76118.26	76222.94	76526.41	76573.94	75345.52	76573.99999
27	76524.95	76135.41	76246.14	76571.86	76621.19	75345.52	76621.25103
28	76568.73	76151.92	76268.73	76617.3	76668.44	75345.52	76668.50207

	Interval#2						
	ARIMA_2	TBATS_2	ETS_2	cubic spline_2	Holt_2	SES_2	HoltWinters_2
1	78186.11	78182.55	78182.48	78174.8	78187.61	78018.3	78187.61684
2	78349.7	78342.17	78342.15	78337.19	78356.05	78018.3	78356.0503
3	78514.85	78496.4	78497.62	78499.58	78524.48	78018.3	78524.48377
4	78686.15	78645.41	78649.01	78661.97	78692.92	78018.3	78692.91723
5	78858.11	78789.39	78796.41	78824.37	78861.35	78018.3	78861.35069
6	79027.74	78928.5	78939.94	78986.76	79029.79	78018.3	79029.78415
7	79193.84	79062.91	79079.7	79149.15	79198.22	78018.3	79198.21762
8	79360.67	79192.78	79215.79	79311.54	79366.66	78018.3	79366.65108
9	79529.7	79318.25	79348.29	79473.94	79535.09	78018.3	79535.08454
10	79700.42	79439.49	79477.32	79636.33	79703.52	78018.3	79703.518
11	79869.91	79556.62	79602.95	79798.72	79871.96	78018.3	79871.95147
12	80037.82	79669.8	79725.28	79961.11	80040.39	78018.3	80040.38493

13	80205.22	79779.15	79844.4	80123.51	80208.83	78018.3	80208.81839
14	80373.84	79884.81	79960.38	80285.9	80377.26	78018.3	80377.25185
15	80543.37	79986.9	80073.31	80448.29	80545.7	78018.3	80545.68532
16	80712.79	80085.53	80183.28	80610.68	80714.13	78018.3	80714.11878
17	80881.25	80180.83	80290.36	80773.08	80882.57	78018.3	80882.55224
18	81049.32	80272.91	80394.62	80935.47	81051	78018.3	81050.9857
19	81217.75	80361.88	80496.14	81097.86	81219.44	78018.3	81219.41917
20	81386.81	80447.84	80594.99	81260.25	81387.87	78018.3	81387.85263
21	81555.93	80530.9	80691.24	81422.65	81556.31	78018.3	81556.28609
22	81724.67	80611.14	80784.96	81585.04	81724.74	78018.3	81724.71955
23	81893.06	80688.68	80876.22	81747.43	81893.17	78018.3	81893.15302
24	82061.56	80763.59	80965.09	81909.82	82061.61	78018.3	82061.58648
25	82230.36	80835.98	81051.61	82072.22	82230.04	78018.3	82230.01994
26	82399.31	80905.91	81135.86	82234.61	82398.48	78018.3	82398.4534
27	82568.09	80973.48	81217.9	82397	82566.91	78018.3	82566.88687
28	82736.68	81038.77	81297.78	82559.39	82735.35	78018.3	82735.32033

	**Interval#3**						
	ARIMA	TBATS	ETS	cubic spline	Holt	SES	HoltWinters
1	82931.01	82915.09	82918.86	82919.31	82924.15	82738.06	82924.19006
2	83129.88	83089.4	83096.3	83103.1	83110.84	82738.06	83110.91269
3	83331.84	83256.98	83269.22	83286.89	83297.53	82738.06	83297.63532
4	83523.91	83418.08	83437.75	83470.69	83484.22	82738.06	83484.35795
5	83714.11	83572.97	83601.99	83654.48	83670.92	82738.06	83671.08058
6	83906.09	83721.87	83762.06	83838.27	83857.61	82738.06	83857.80321
7	84104.91	83865.02	83918.05	84022.06	84044.3	82738.06	84044.52584
8	84303.67	84002.64	84070.07	84205.85	84230.99	82738.06	84231.24847
9	84499.82	84134.94	84218.23	84389.65	84417.68	82738.06	84417.9711
10	84692.18	84262.14	84362.62	84573.44	84604.38	82738.06	84604.69373
11	84885.81	84384.42	84503.33	84757.23	84791.07	82738.06	84791.41636
12	85081.78	84501.98	84640.47	84941.02	84977.76	82738.06	84978.13899
13	85279.45	84614.99	84774.12	85124.81	85164.45	82738.06	85164.86162
14	85475.52	84723.64	84904.36	85308.6	85351.15	82738.06	85351.58425
15	85669.98	84828.1	85031.3	85492.4	85537.84	82738.06	85538.30688
16	85864	84928.52	85155.01	85676.19	85724.53	82738.06	85725.02951
17	86059.47	85025.06	85275.56	85859.98	85911.22	82738.06	85911.75214
18	86255.83	85117.87	85393.06	86043.77	86097.91	82738.06	86098.47477
19	86451.98	85207.1	85507.56	86227.56	86284.61	82738.06	86285.1974
20	86647.05	85292.88	85619.15	86411.35	86471.3	82738.06	86471.92003
21	86841.78	85375.35	85727.91	86595.15	86657.99	82738.06	86658.64266
22	87036.95	85454.63	85833.89	86778.94	86844.68	82738.06	86845.36529
23	87232.81	85530.85	85937.18	86962.73	87031.37	82738.06	87032.08792
24	87428.66	85604.12	86037.85	87146.52	87218.07	82738.06	87218.81055
25	87624.08	85674.57	86135.95	87330.31	87404.76	82738.06	87405.53318
26	87819.14	85742.29	86231.55	87514.11	87591.45	82738.06	87592.25581
27	88014.36	85807.4	86324.73	87697.9	87778.14	82738.06	87778.97844
28	88209.91	85869.99	86415.53	87881.69	87964.83	82738.06	87965.70107

Table S6: Riyadh actual cases

	Interval#1	Interval#2	Interval#3
DAY	Confirmed	Confirmed	Confirmed
1	75389	78193	82917
2	75435	78369	83081
3	75501	78537	83255
4	75559	78698	83430
5	75621	78865	83609
6	75688	79019	83751
7	75741	79170	83888
8	75827	79339	84032
9	75910	79504	84189
10	75998	79667	84348
11	76076	79838	84520
12	76158	80015	84685
13	76233	80189	84869
14	76311	80352	85028
15	76395	80538	85221
16	76480	80718	85425
17	76569	80902	85638
18	76674	81081	85864
19	76767	81248	86085
20	76866	81390	86302
21	76997	81526	86530
22	77121	81660	86764
23	77247	81822	87002
24	77381	82009	87340
25	77535	82192	87660
26	77688	82380	87979
27	77852	82548	88268
28	78020	82740	88631

Supplemental R code

```
#install packages
library(forecast)

#Loading the dataset and defining the time series object
dt <- read.csv("KSA_ts_covid19.csv")
ksa_ts <- ts(dt$Confirmed, frequency = 365.25, start = c(2020, 84))

# Splitting the time series into training and testing sets
wholeset <- ts_split(ts.obj = ksa_ts, sample.out = 28)
#Training set
tts <- wholeset$train
#Testing set
testing28 <- wholeset$test

#Automatic ARIMA
model_arima <- auto.arima(tts, trace = TRUE)
forecast_arima <- forecast(model_arima,  h = 28)
accuracy(forecast _arima,testing28)

#TBATS
model_tbats <- tbats(tts)
forecast _tbats <- forecast :: forecast(model_tbats, h = 28)
accuracy(forecast _tbats,testing28)

#ETS
model_ets <- ets(tts)
forecast_ets = forecast(model_ets, h=28)
accuracy(forecast_ets, testing28)

#Cubic smoothing spline
forecast _spline <- splinef(tts, h=28)
accuracy(forecast_spline, testing28)

#Holt
holtts <- holt(tts, h = 28)
accuracy(holtts, testing28)

#HoltWinters
model_hw <- HoltWinters(tts, gamma = FALSE)
hwforecast <- forecast(model_hw, h=28 )
accuracy(hwforecast,testing28 )

#SES
sests <- ses(tts,  h = 28)
accuracy(sests, testing28)

#The analysis was repeated for all periods after incorporating the historical data of the previous period(s).
```
