# Supplementary material for: Empirical Evaluation of Alternative Time-Series Models for COVID-19 Forecasting in Saudi Arabia
Source: Int J Environ Res Public Health. 2021 Aug 16;18(16):8660. doi: 10.3390/ijerph18168660 (PMC8393561; doi:10.3390/ijerph18168660)

Forecasted cases for all models with the prediction periods

[The dark blue areas are the 80% prediction periods, and the light blue are the 95% prediction periods]

1- confirmed cases in Saudi Arabia

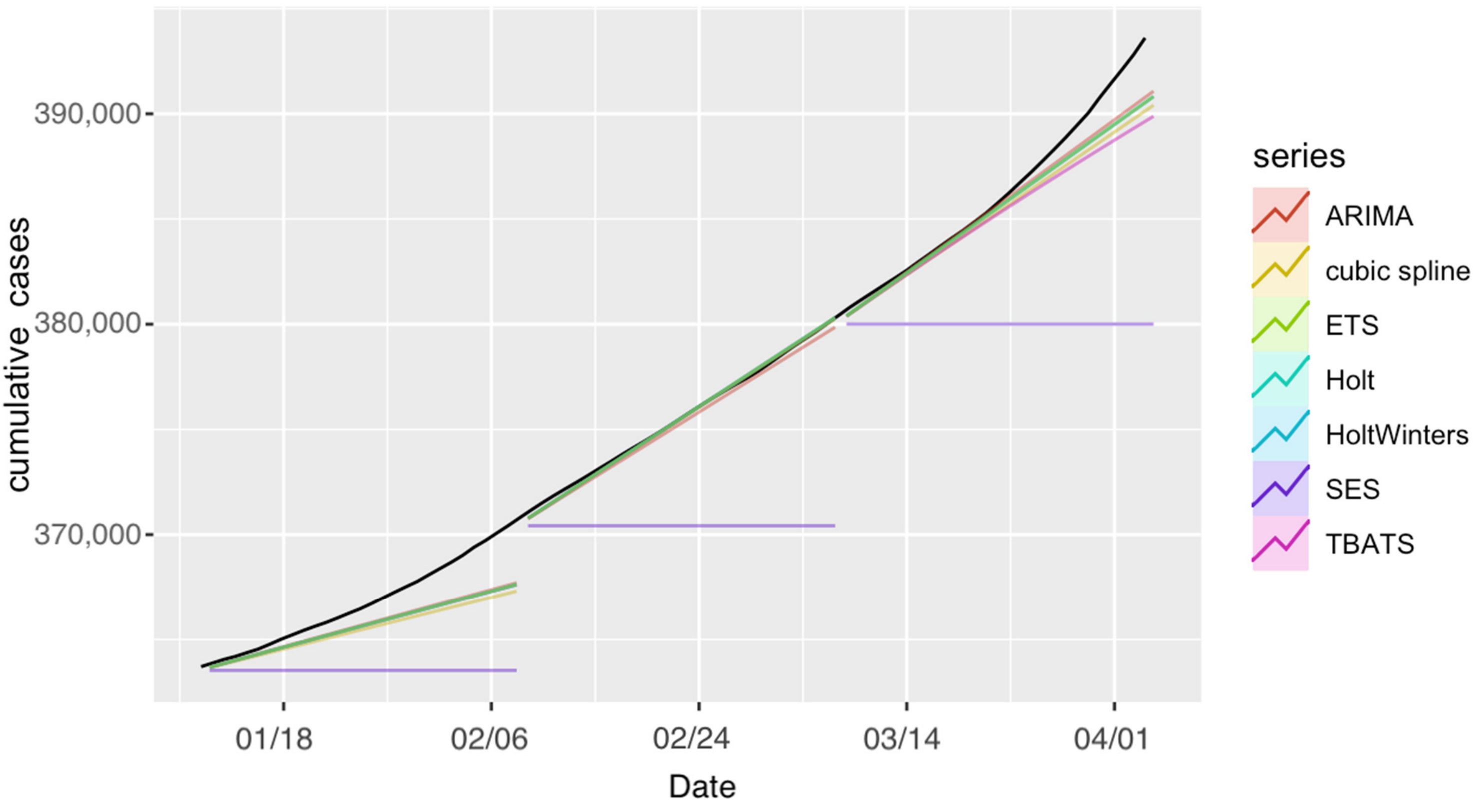

The left side of this figure is trimmed to have better visualization wherein we have forecasting periods

(a)ARIMA

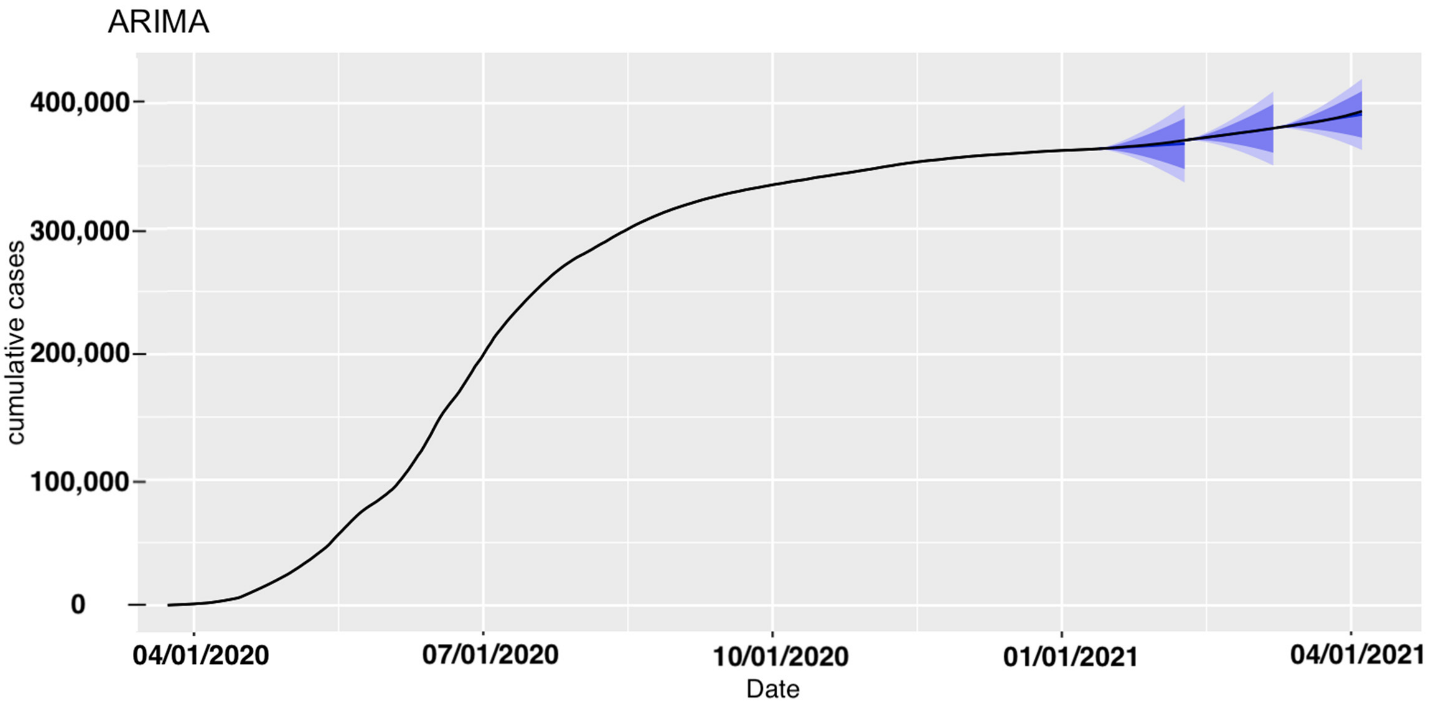

(b)TBATS

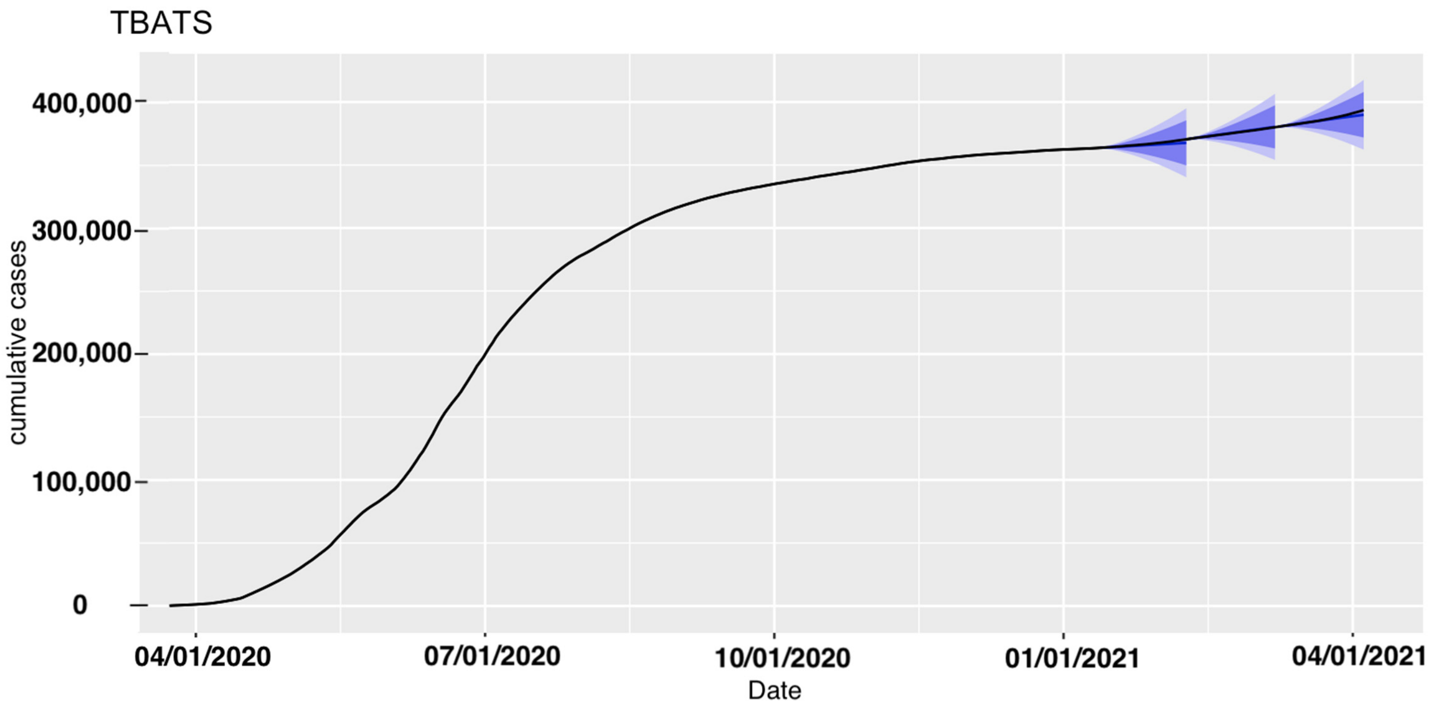

(c)ETS

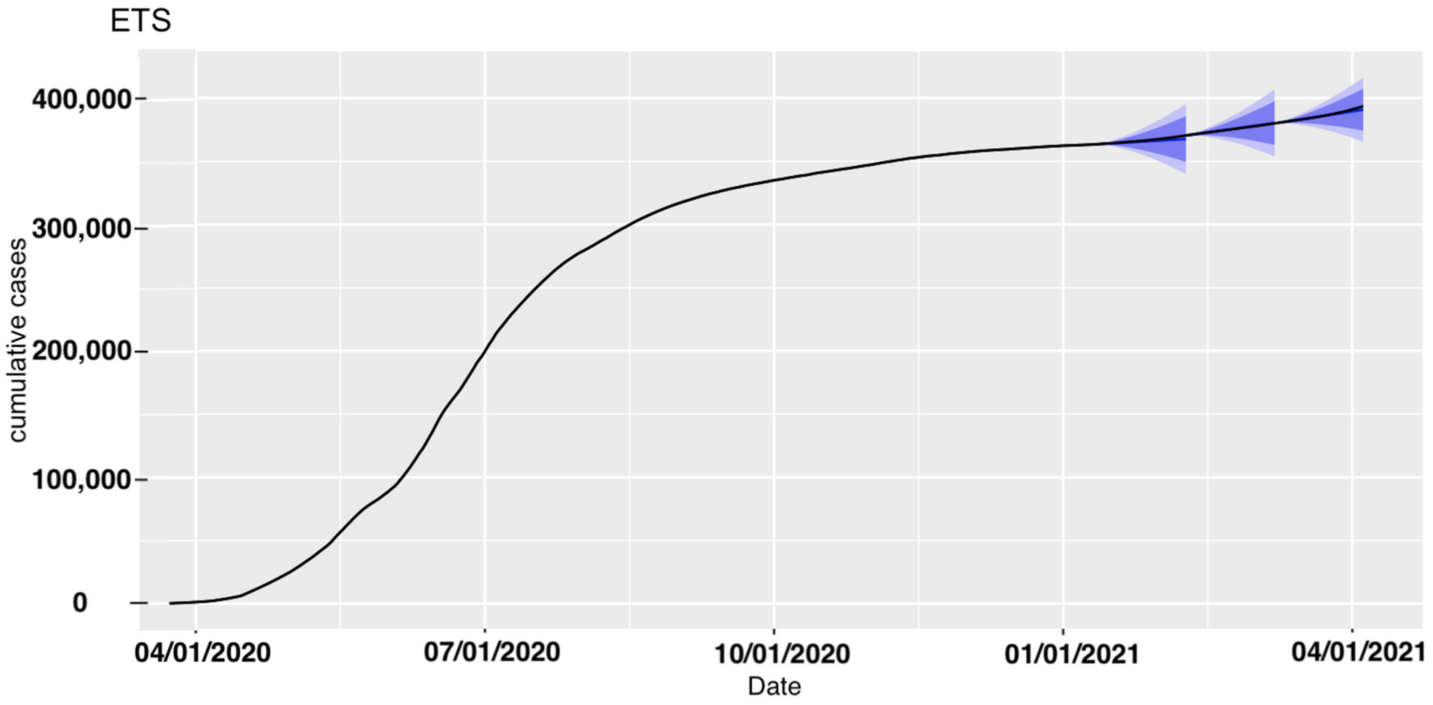

ETS
400,000
300,000
200,000
100,000
0
cumulative cases
04/01/2020
07/01/2020
10/01/2020
01/01/2021
04/01/2021
Date

(d)Holt

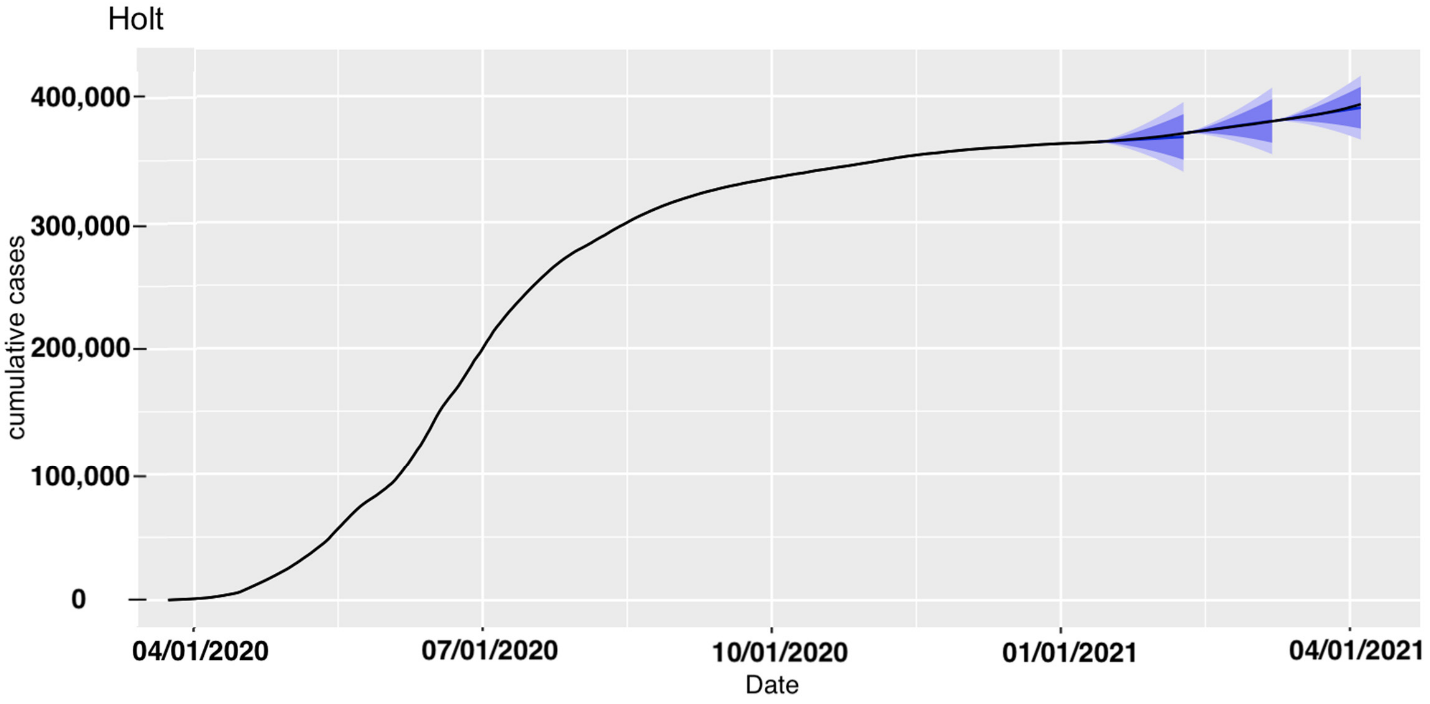

Holt
400,000
300,000
200,000
100,000
0
cumulative cases
04/01/2020
07/01/2020
10/01/2020
01/01/2021
04/01/2021
Date

(e)SES

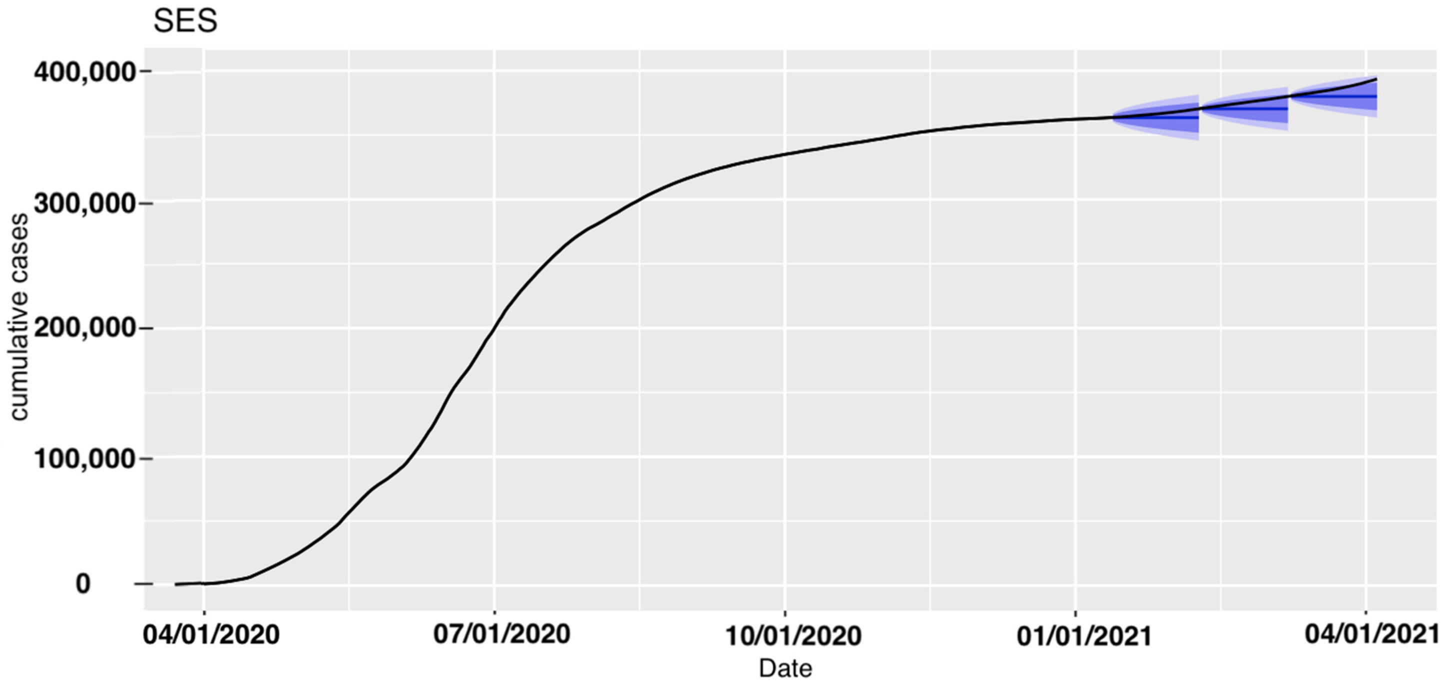

(f)HoltWinters

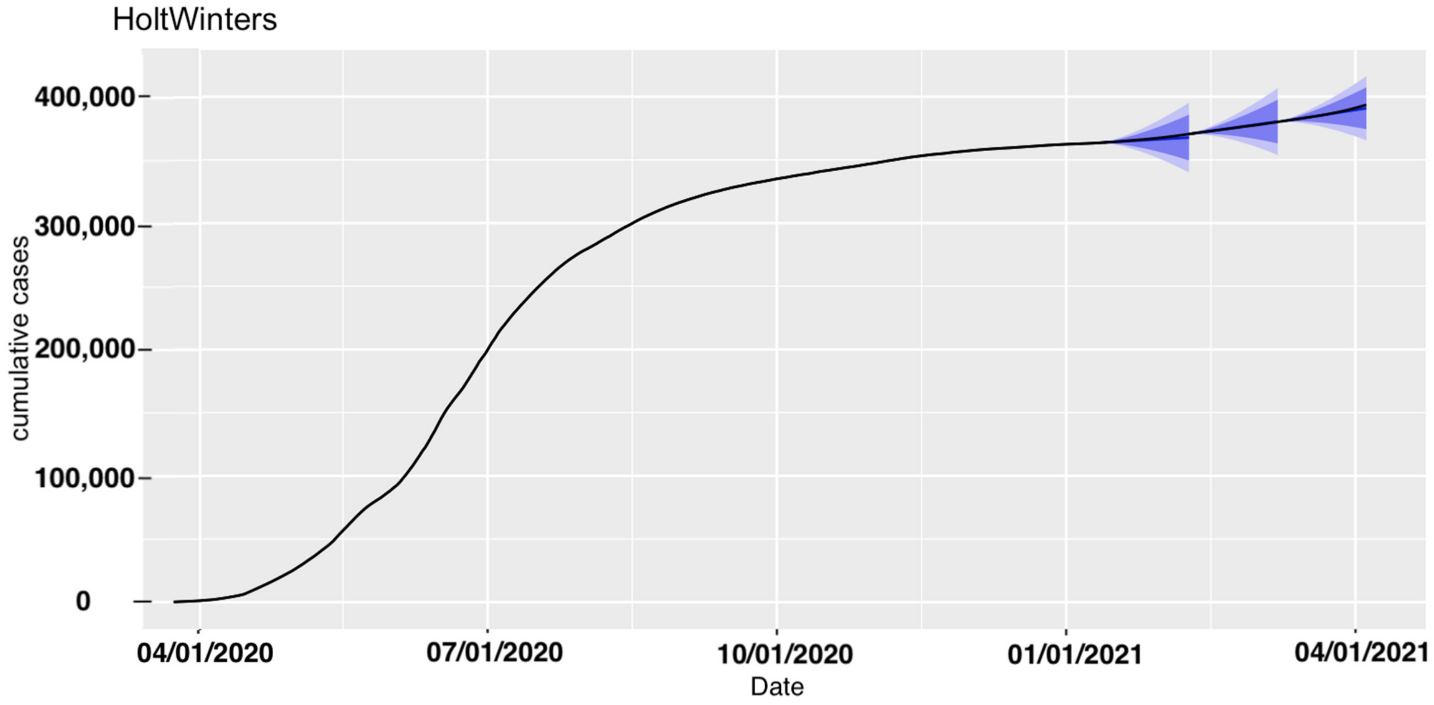

(g)cubic spline

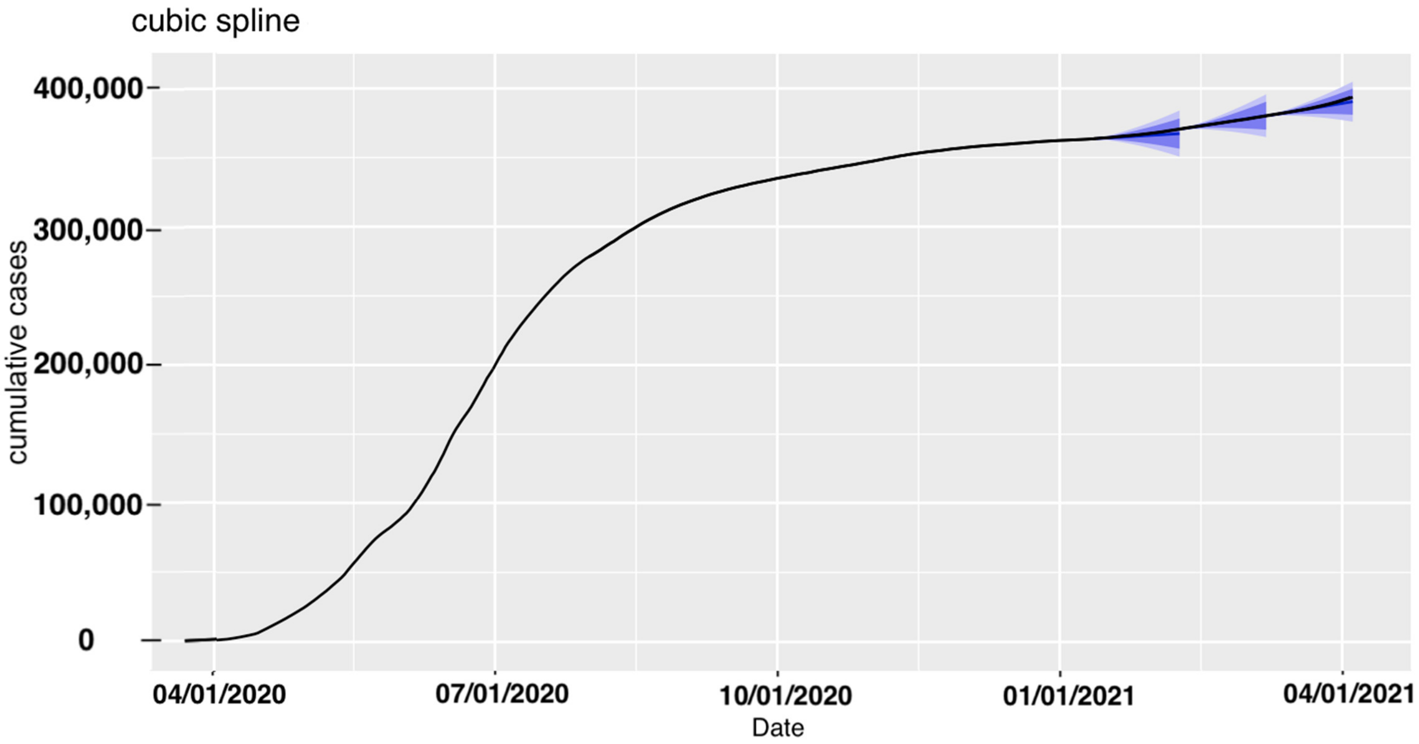

2- recovered cases in Saudi Arabia

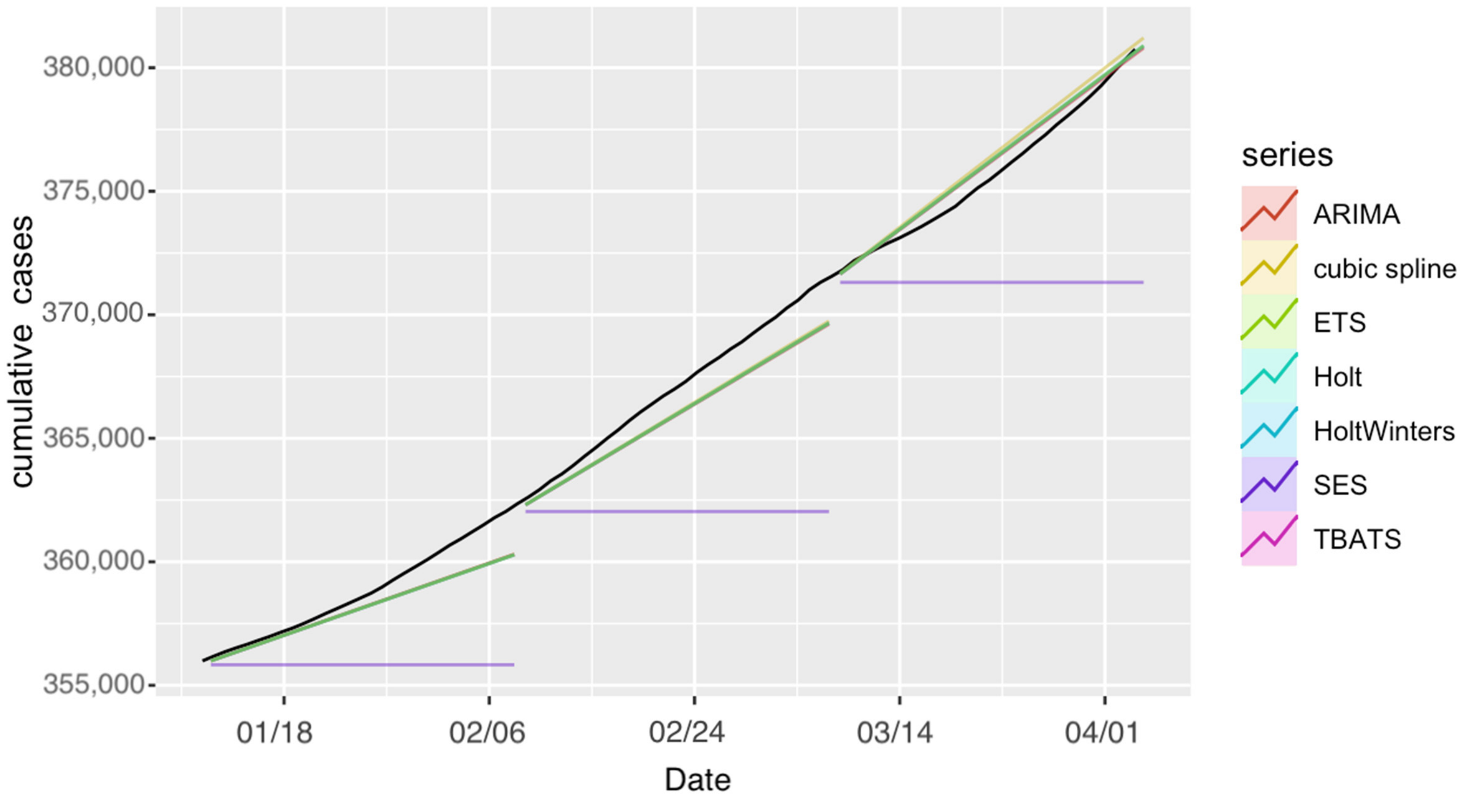

(a)ARIMA

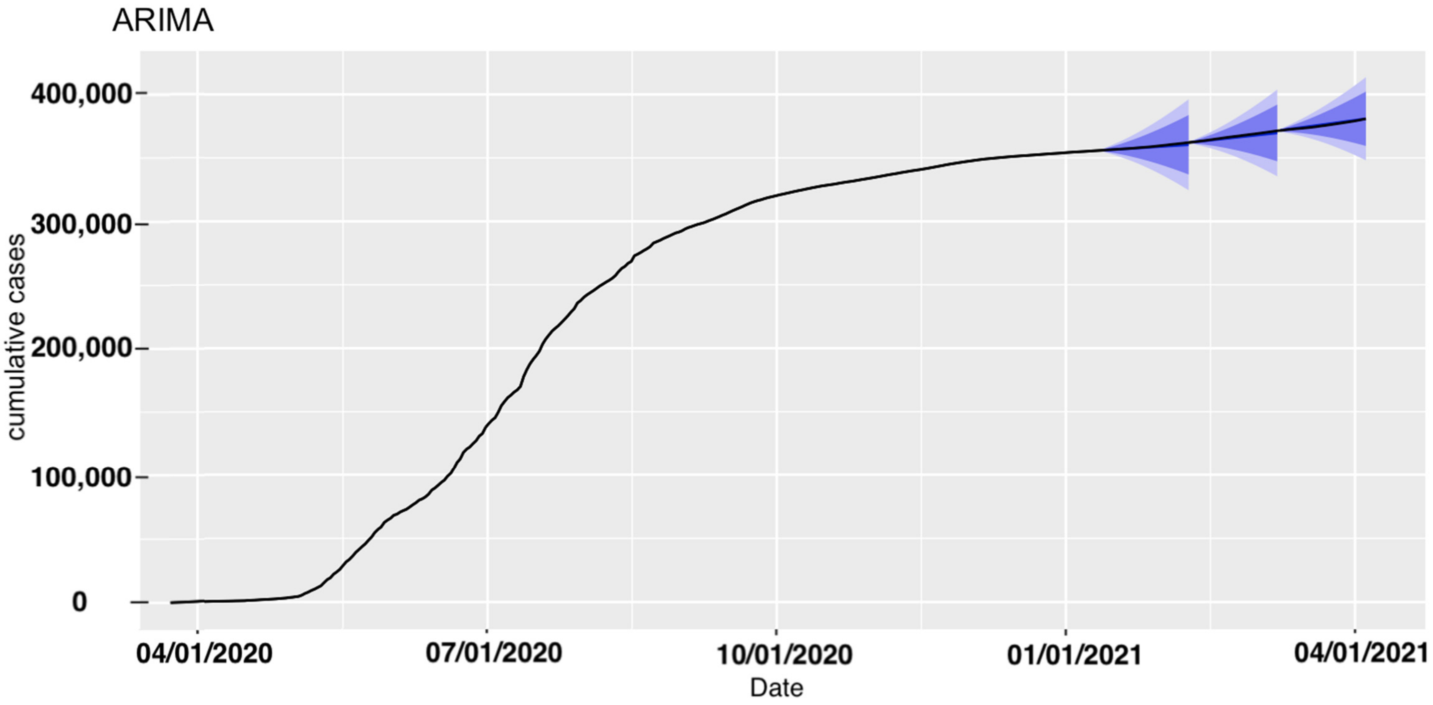

(b)TBATS

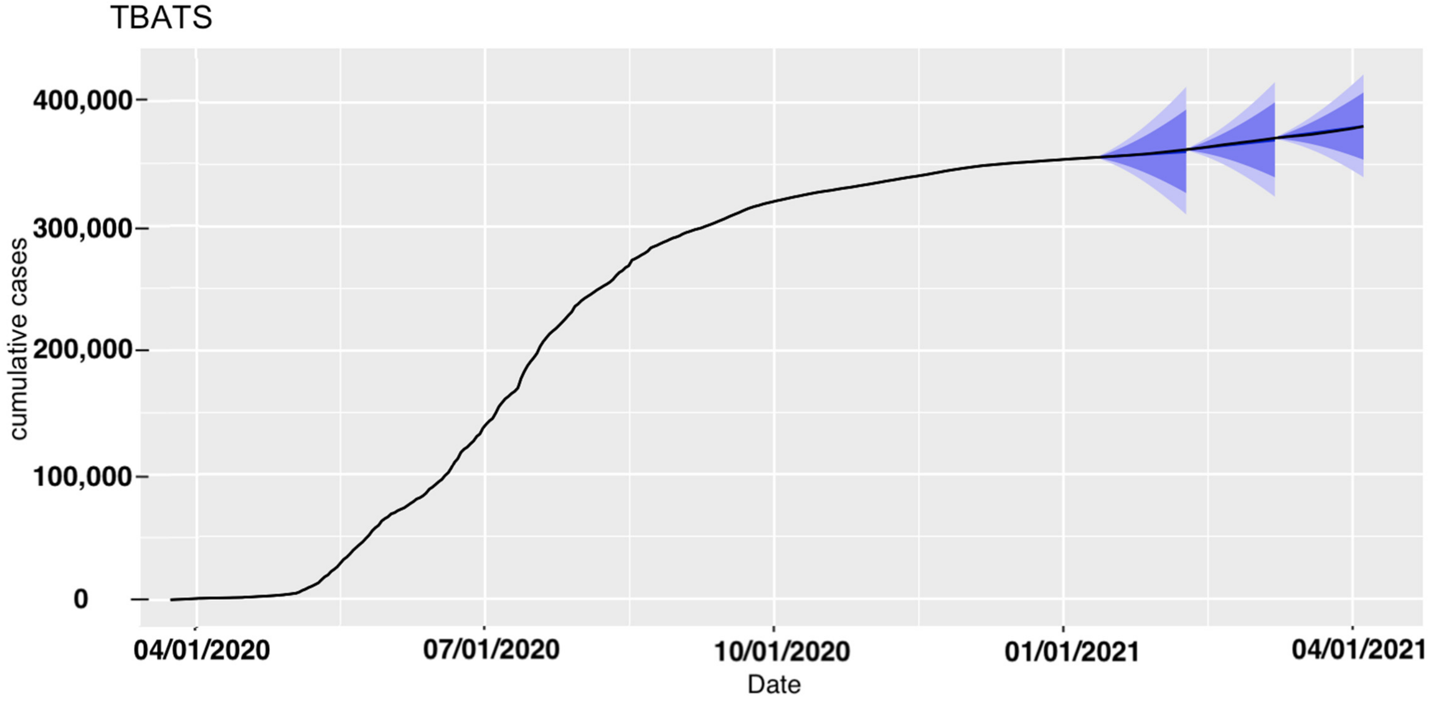

(c)ETS

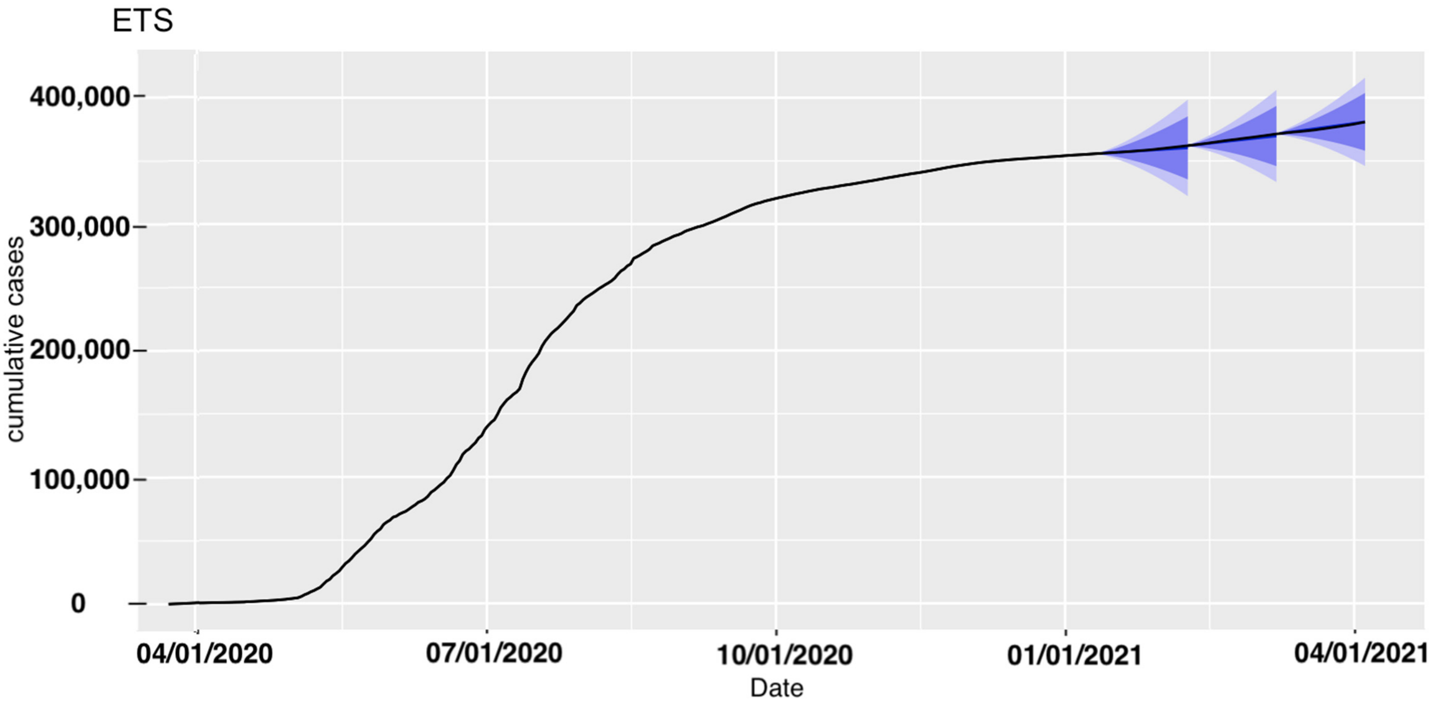
ETS
400,000
300,000
200,000
100,000
0
cumulative cases
04/01/2020
07/01/2020
10/01/2020
01/01/2021
04/01/2021
Date

(d)Holt

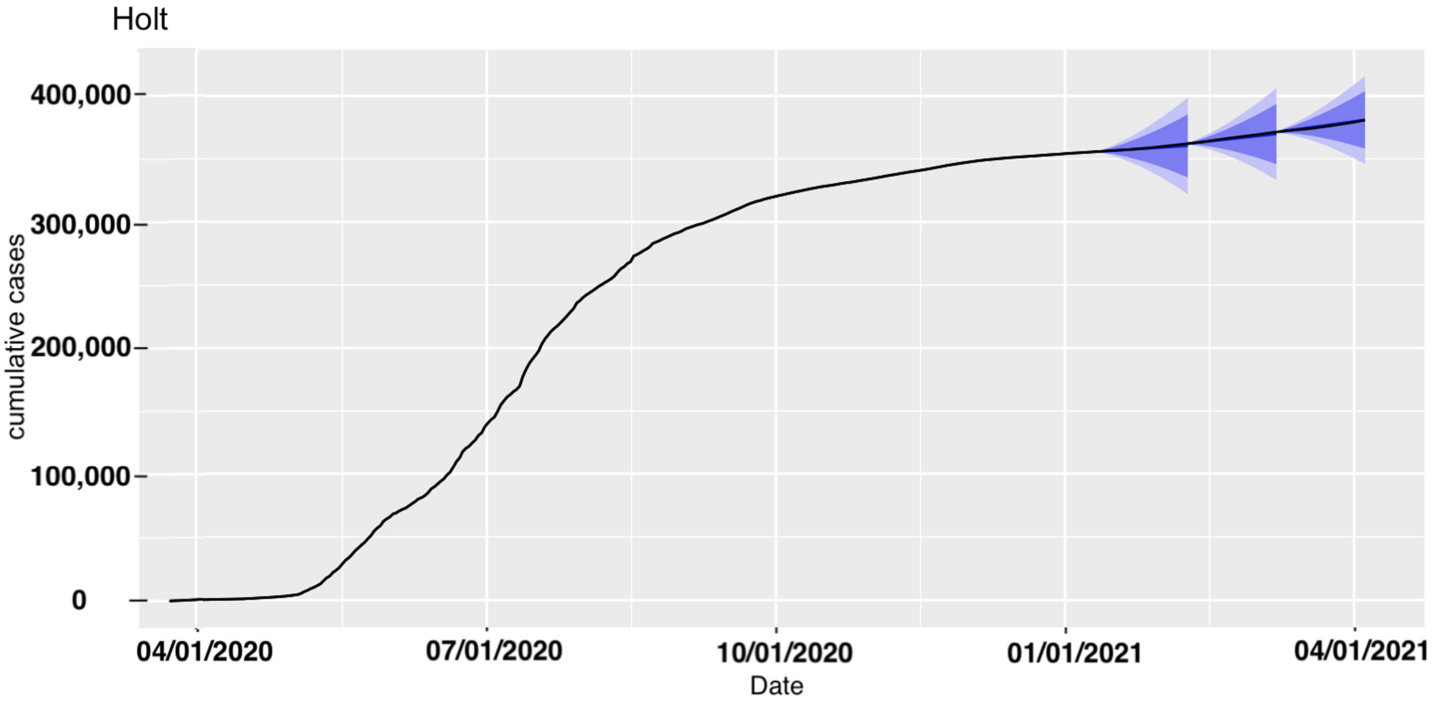
Holt
400,000
300,000
200,000
100,000
0
cumulative cases
04/01/2020
07/01/2020
10/01/2020
01/01/2021
04/01/2021
Date

(e)SES

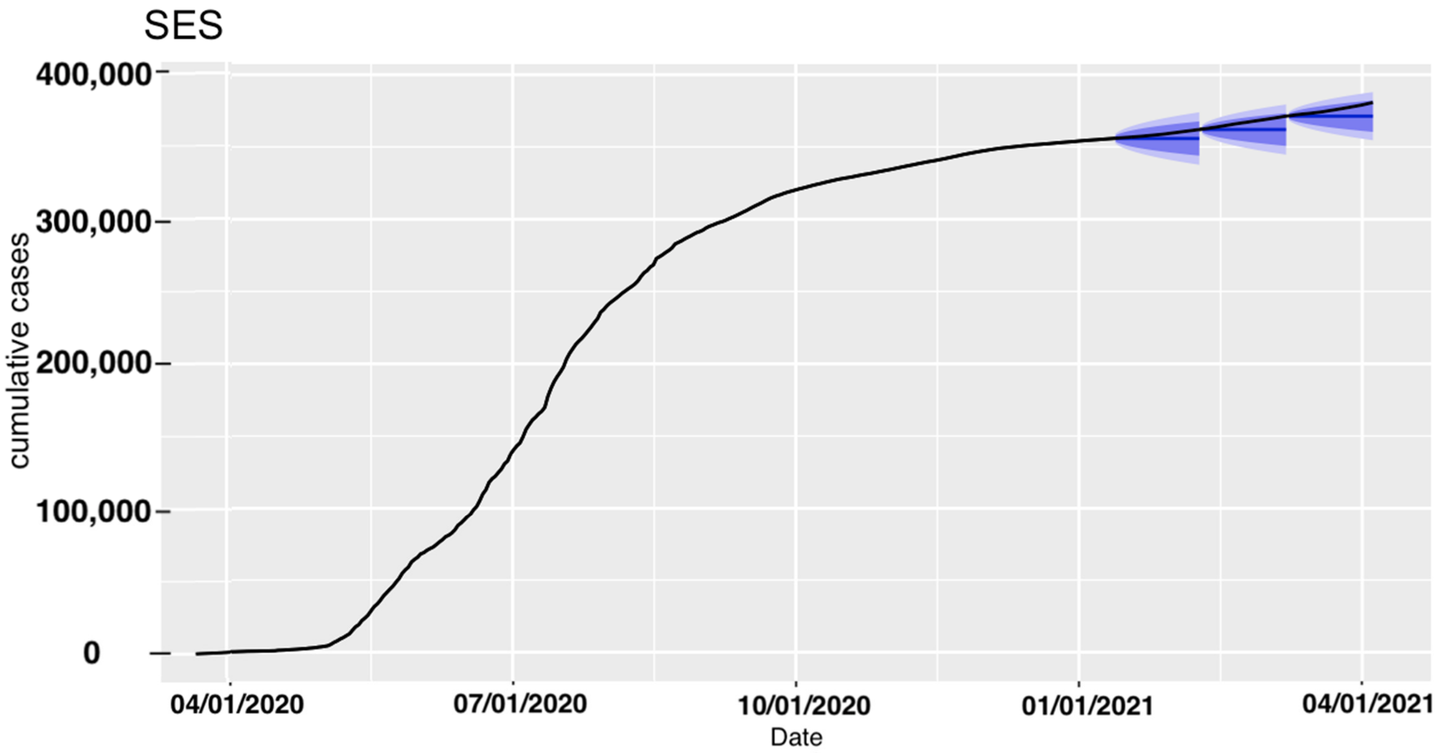

(f)HoltWinters

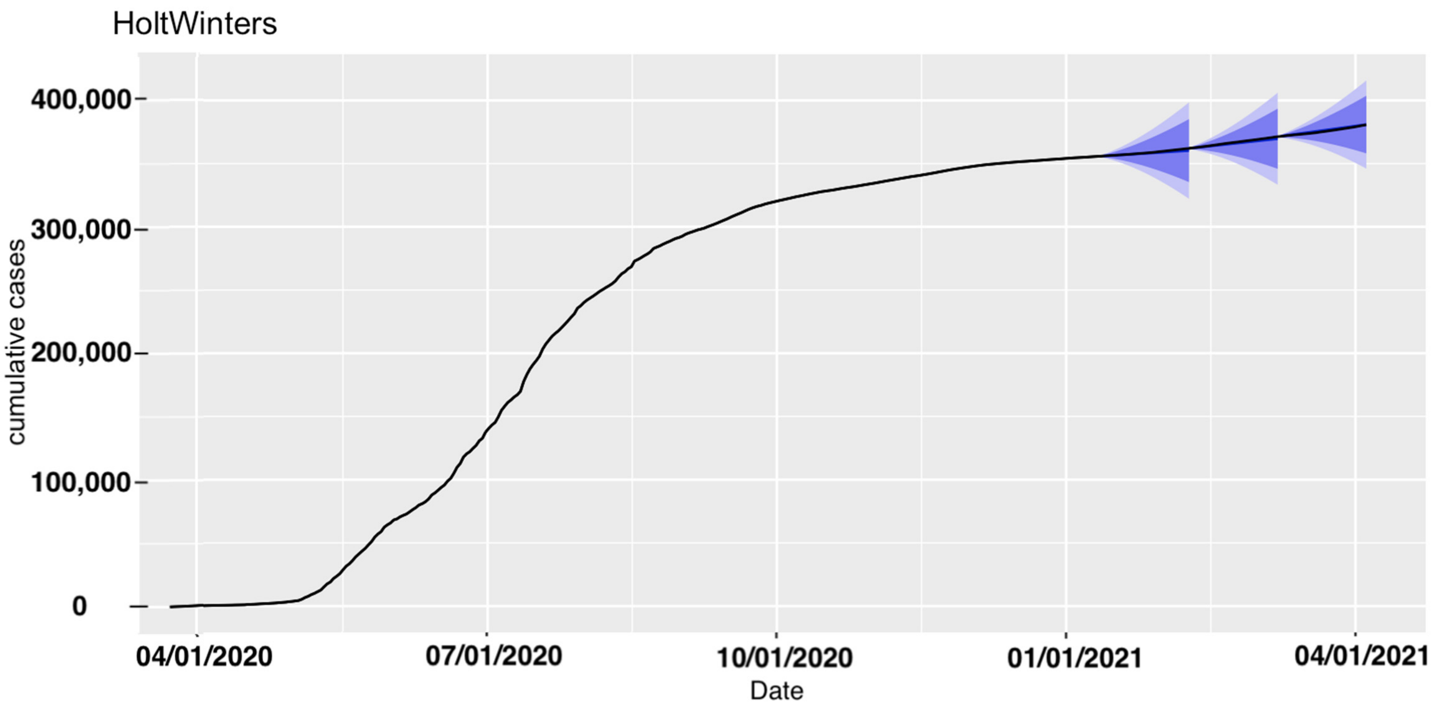

(g)cubic spline

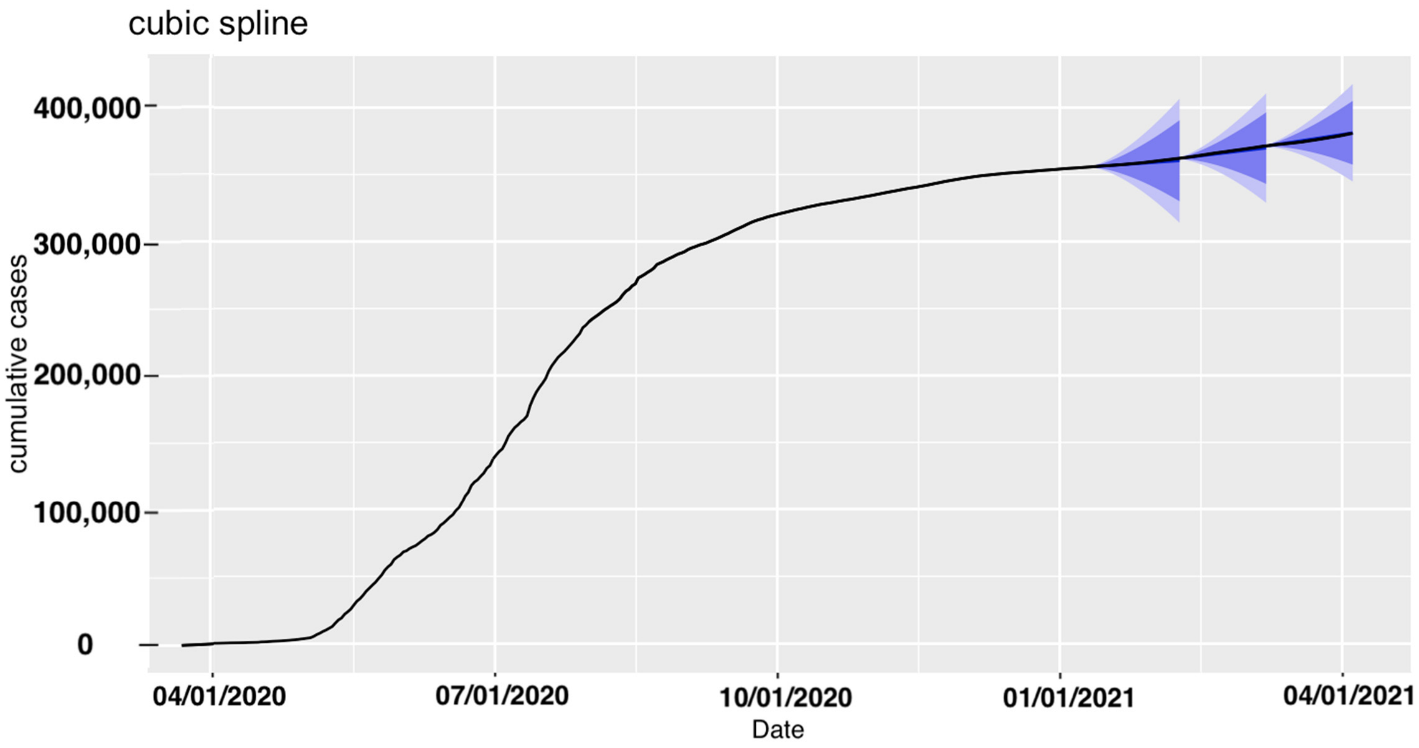

3- deaths in Saudi Arabia

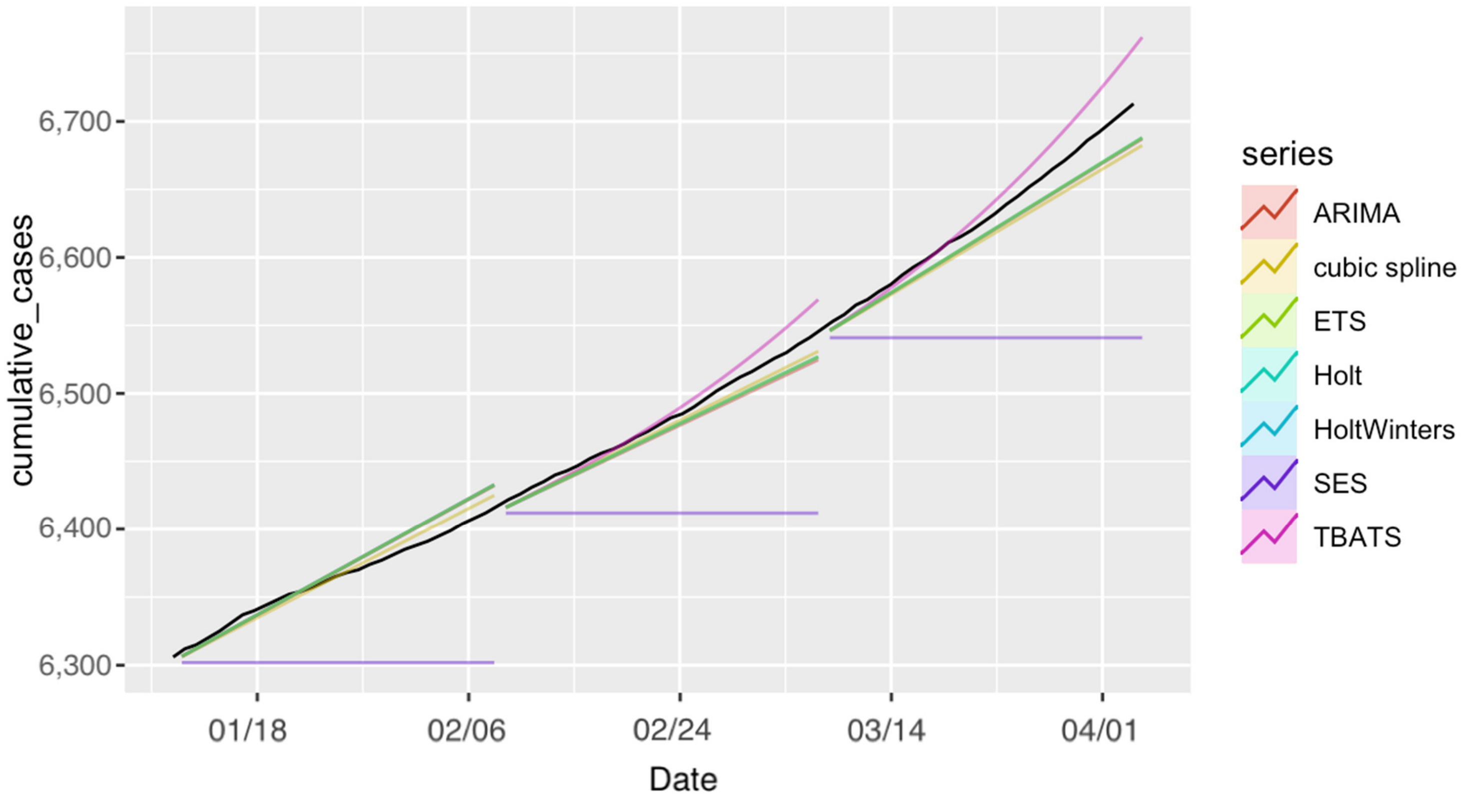

(a)ARIMA

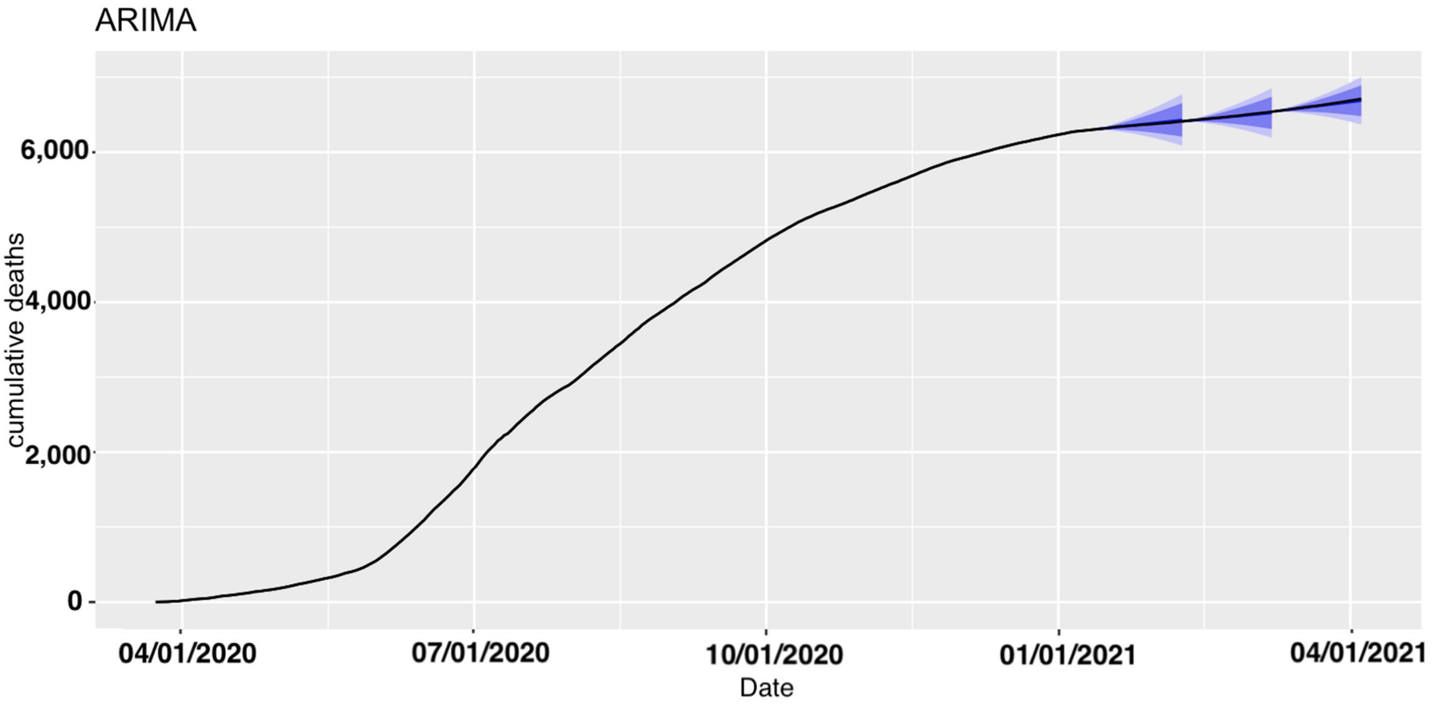

(b)TBATS

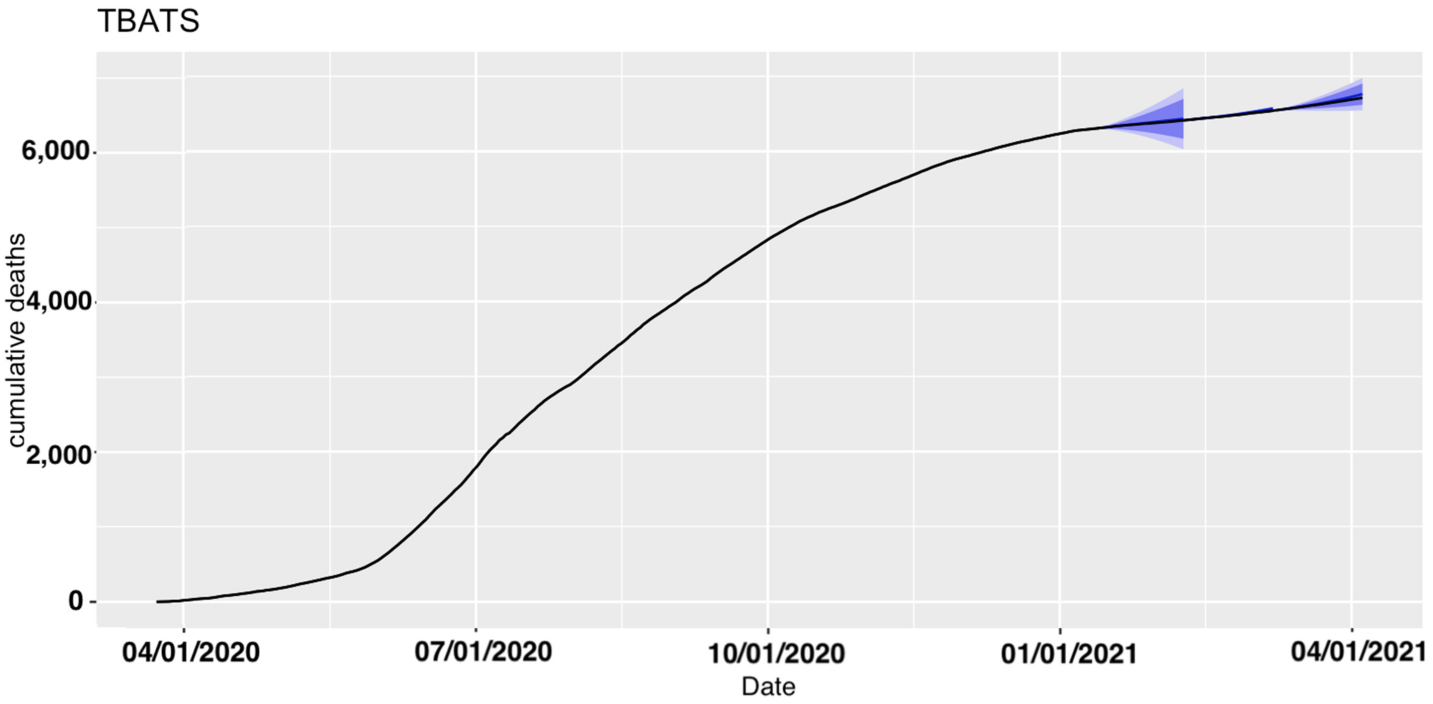

(c)ETS

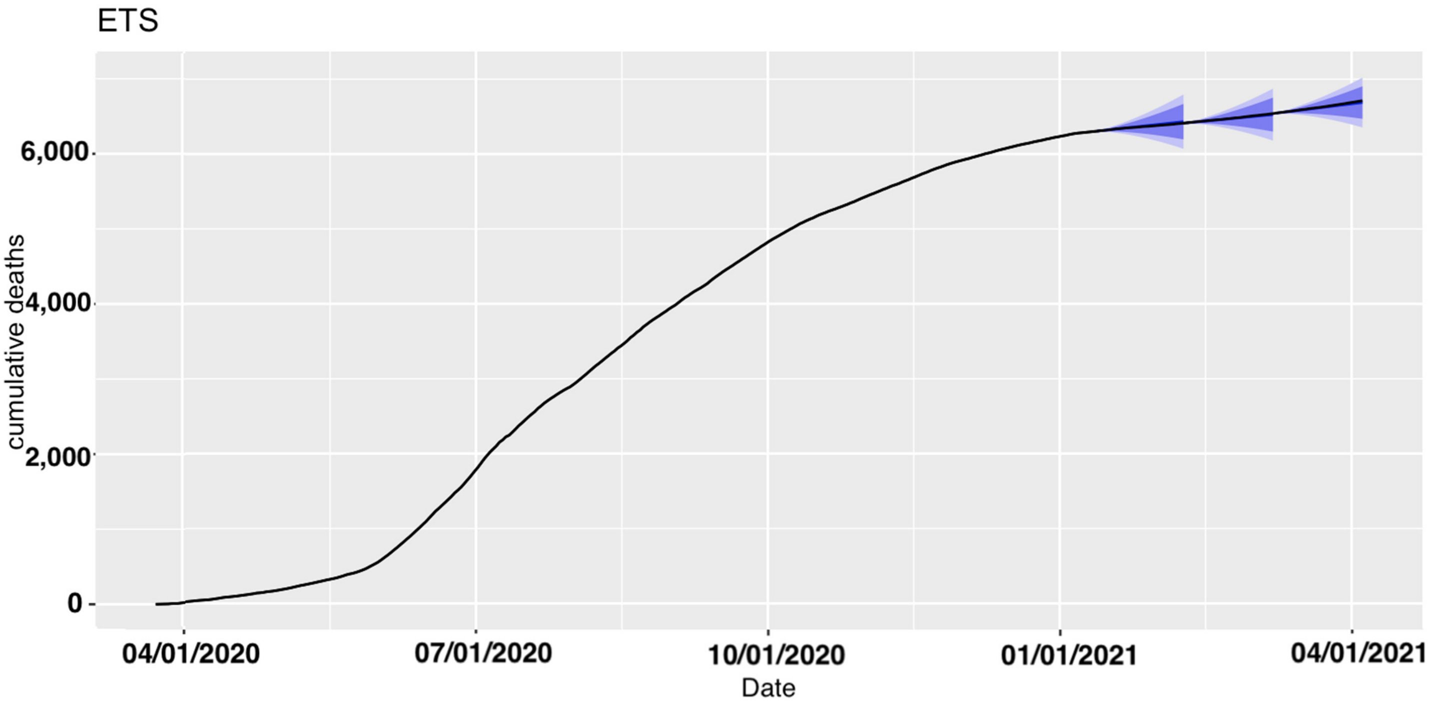
ETS
6,000
4,000
2,000
0
cumulative deaths
04/01/2020
07/01/2020
10/01/2020
01/01/2021
04/01/2021
Date

(d)Holt

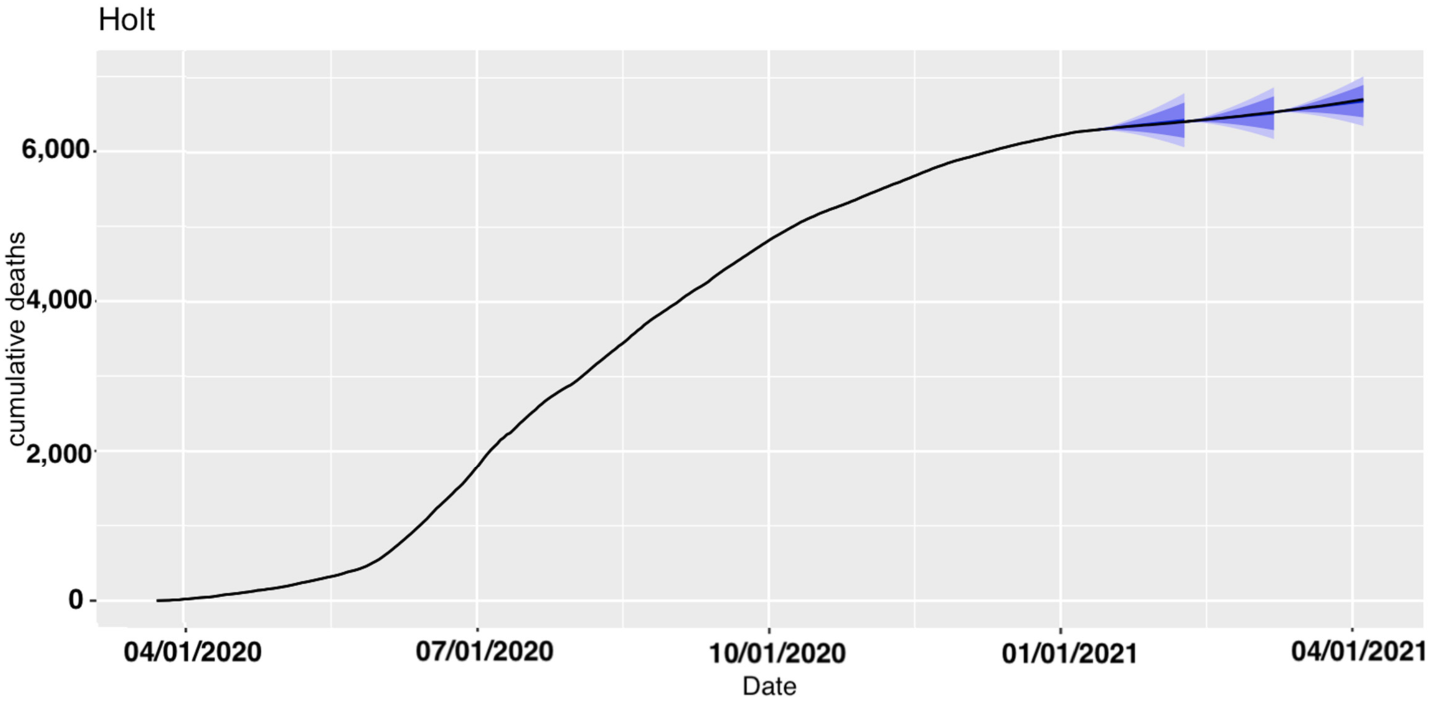
Holt
6,000
4,000
2,000
0
cumulative deaths
04/01/2020
07/01/2020
10/01/2020
01/01/2021
04/01/2021
Date

(e)SES

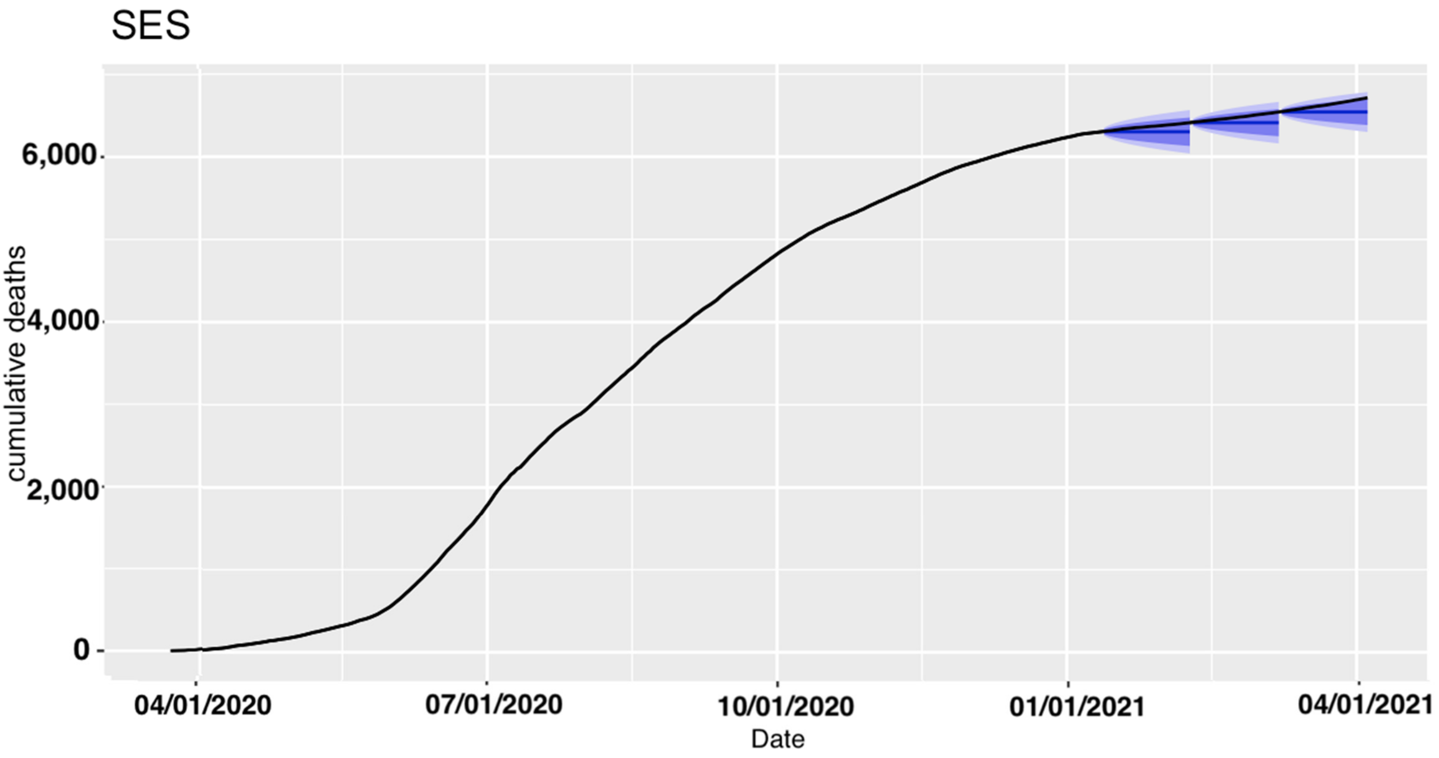

(f)HoltWinters

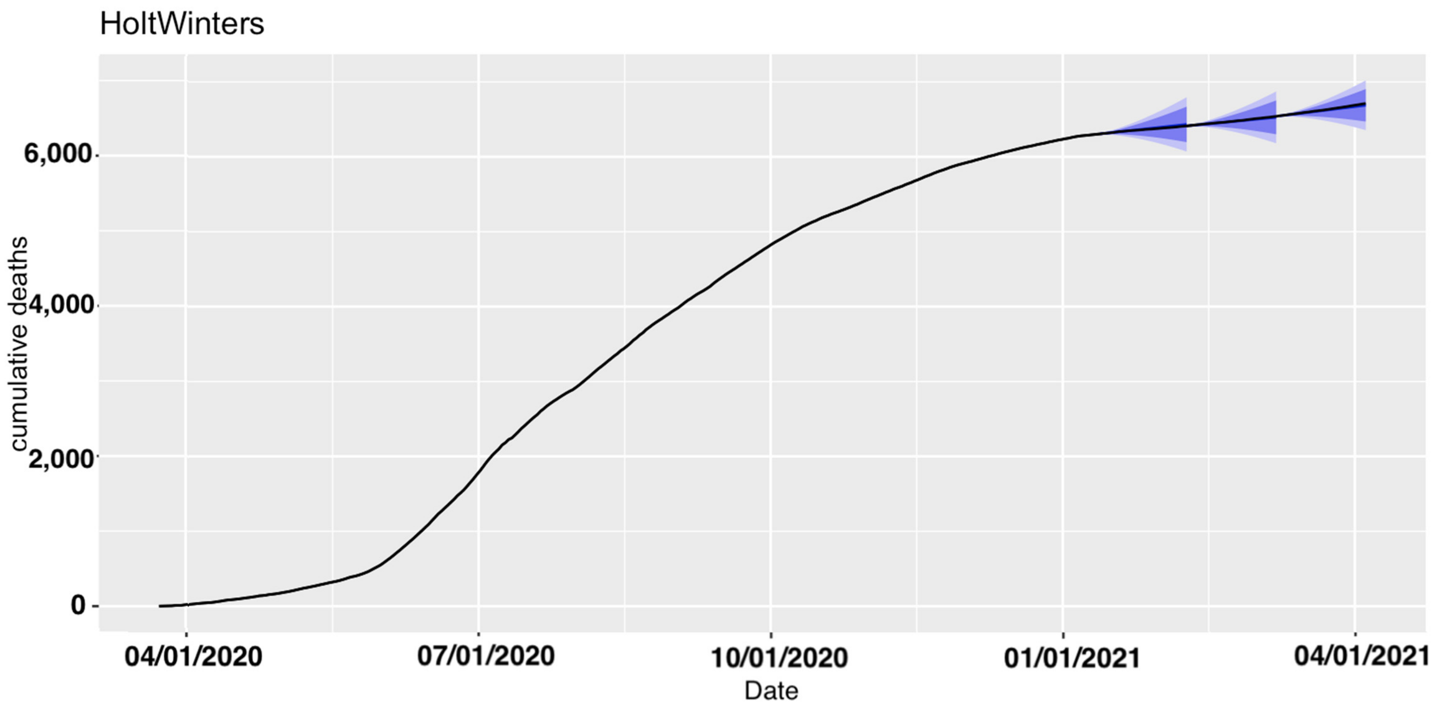

(g)cubic spline

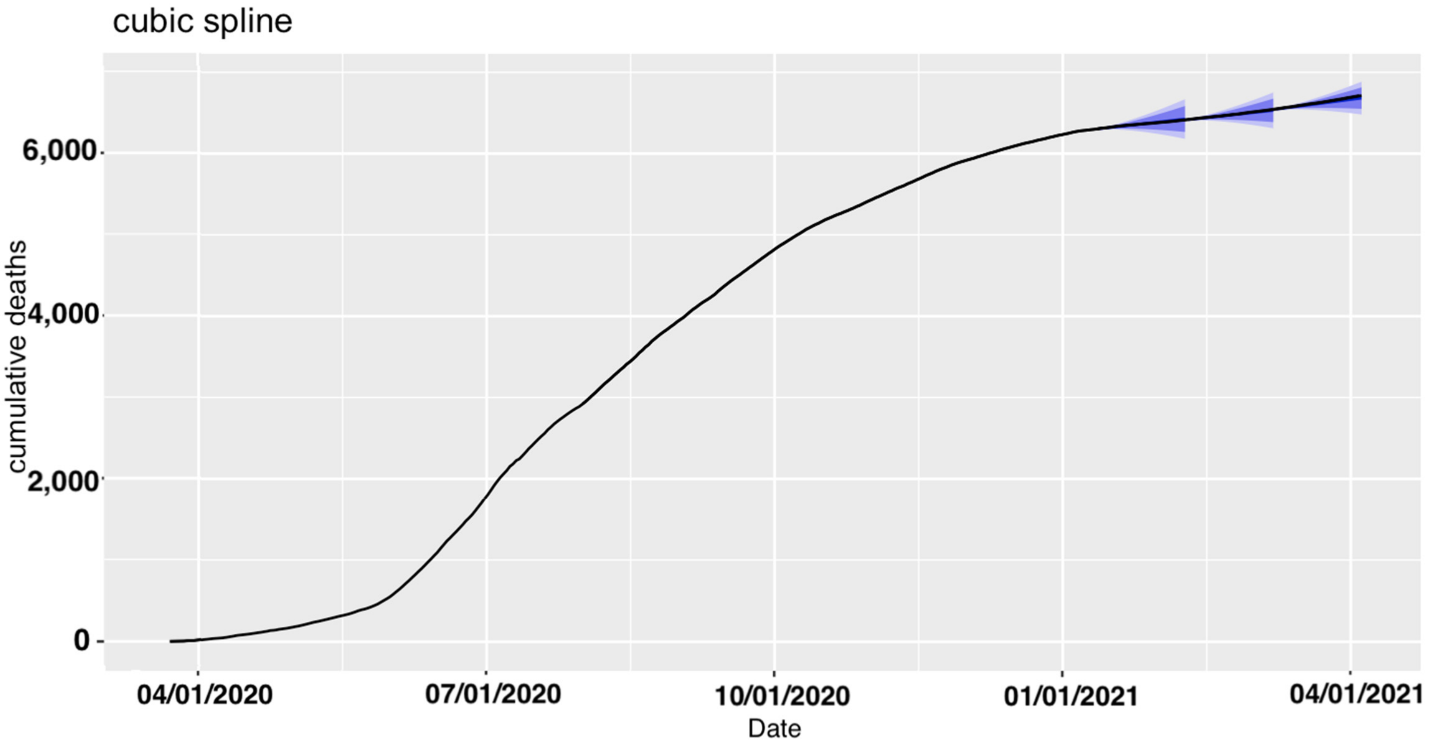

4- confirmed cases in Riyadh

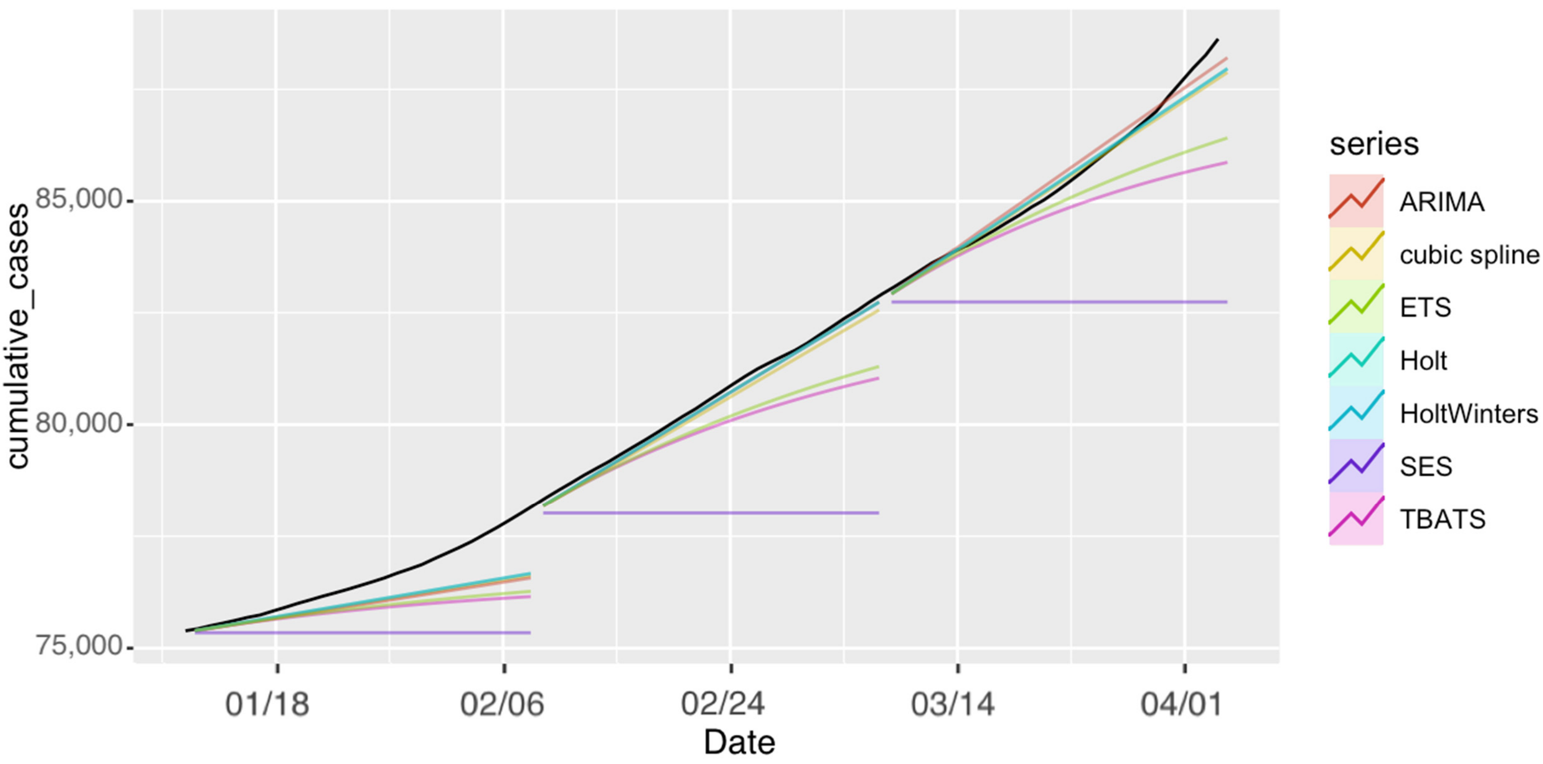

(a)ARIMA

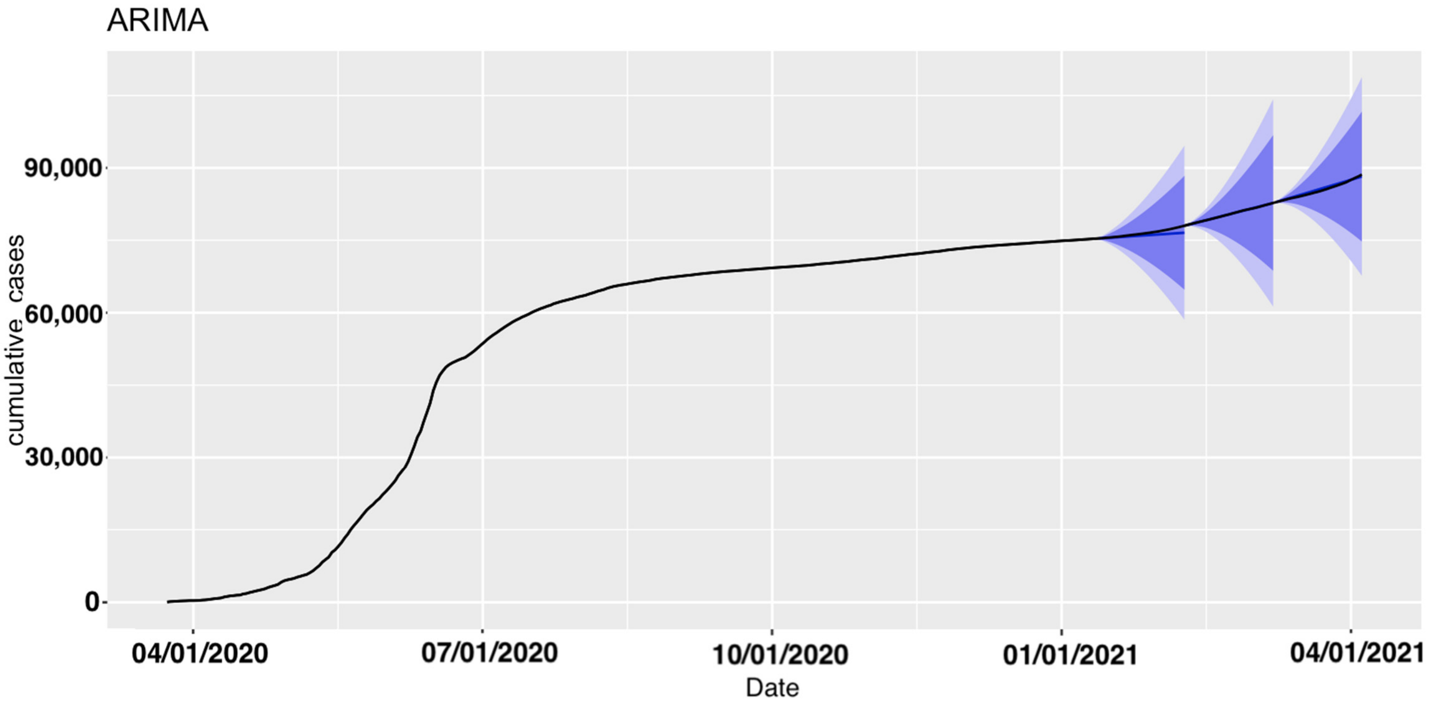

(b)TBATS

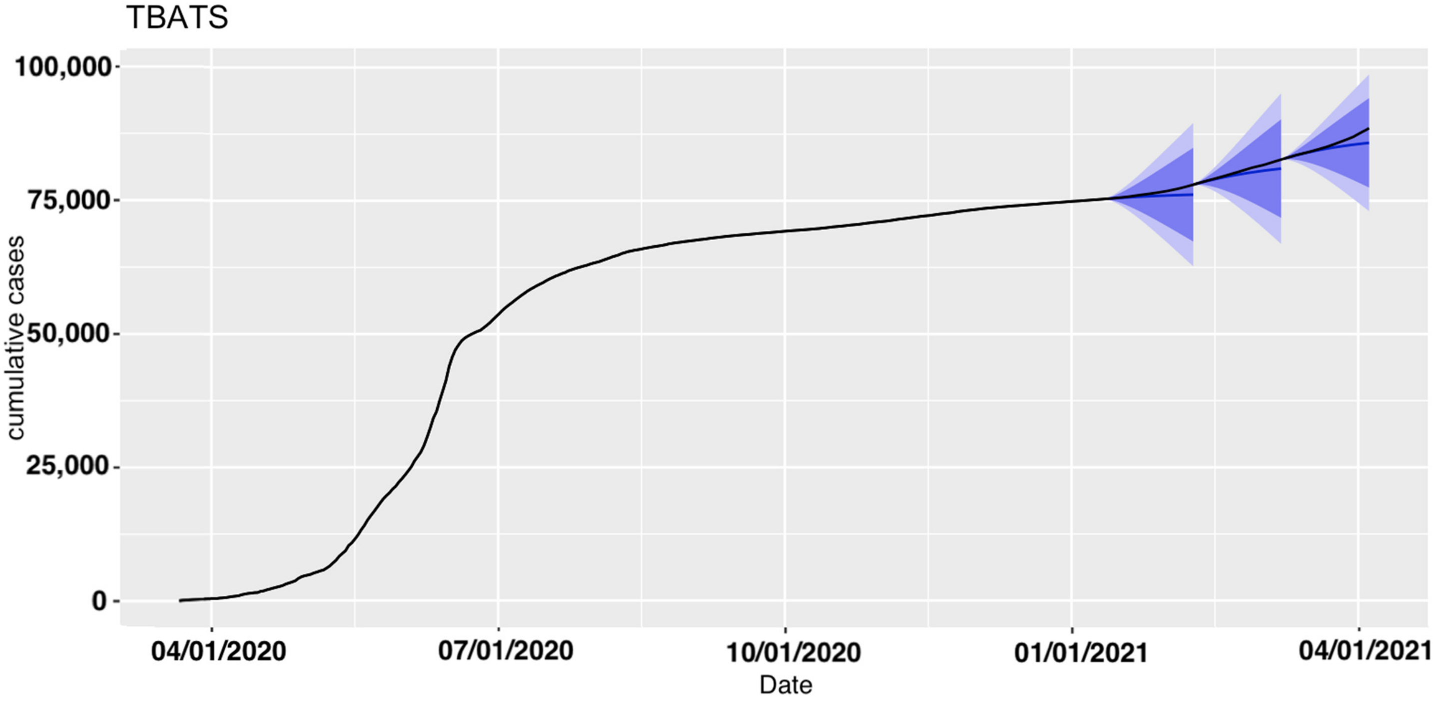

(c)ETS

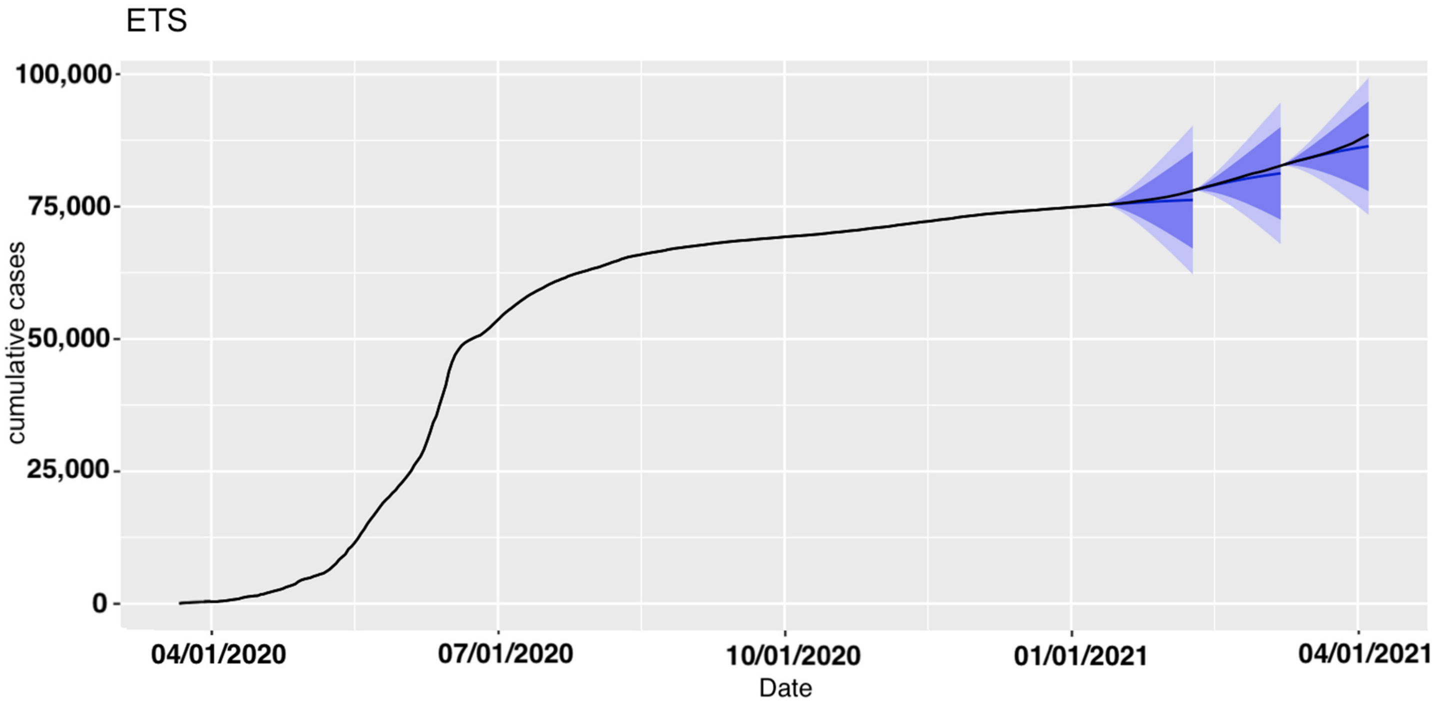
ETS
100,000
75,000
50,000
25,000
0
cumulative cases
04/01/2020
07/01/2020
10/01/2020
01/01/2021
04/01/2021
Date

(d)Holt

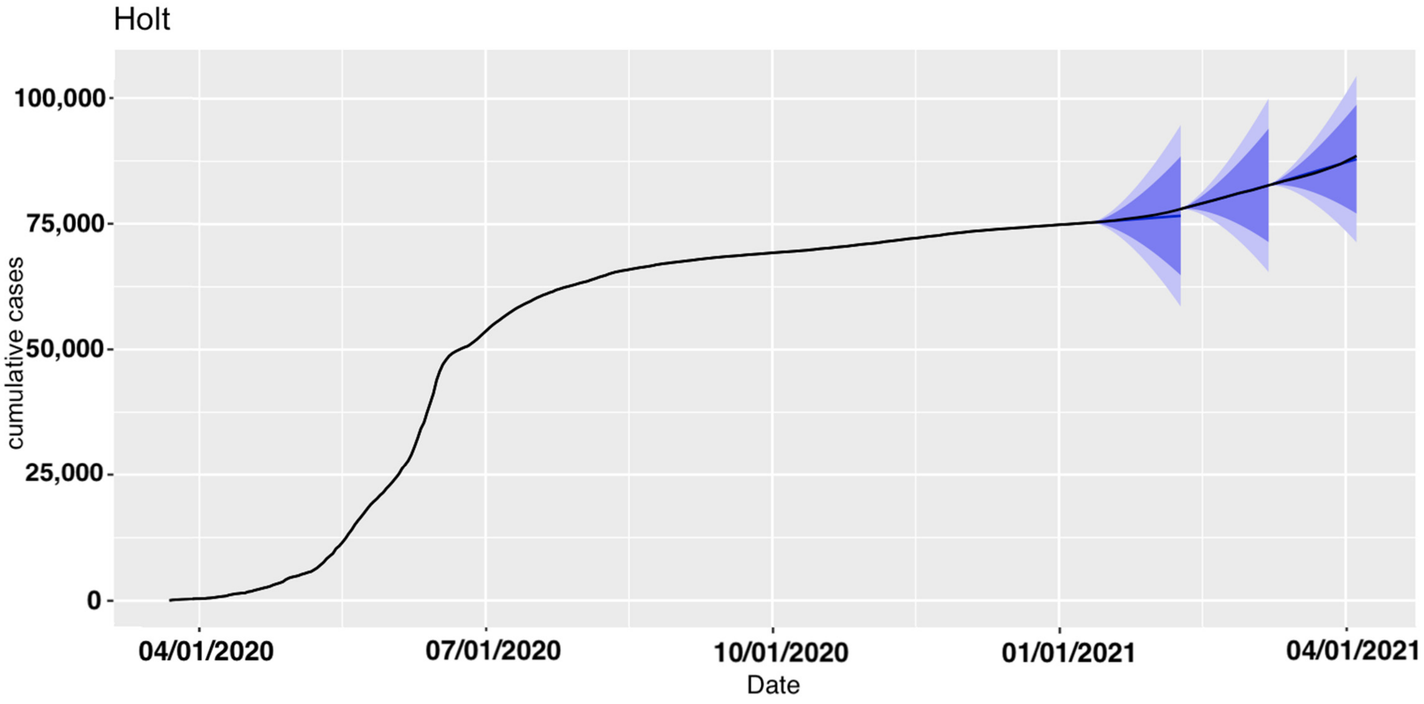
Holt
100,000
75,000
50,000
25,000
0
cumulative cases
04/01/2020
07/01/2020
10/01/2020
01/01/2021
04/01/2021
Date

(e)SES

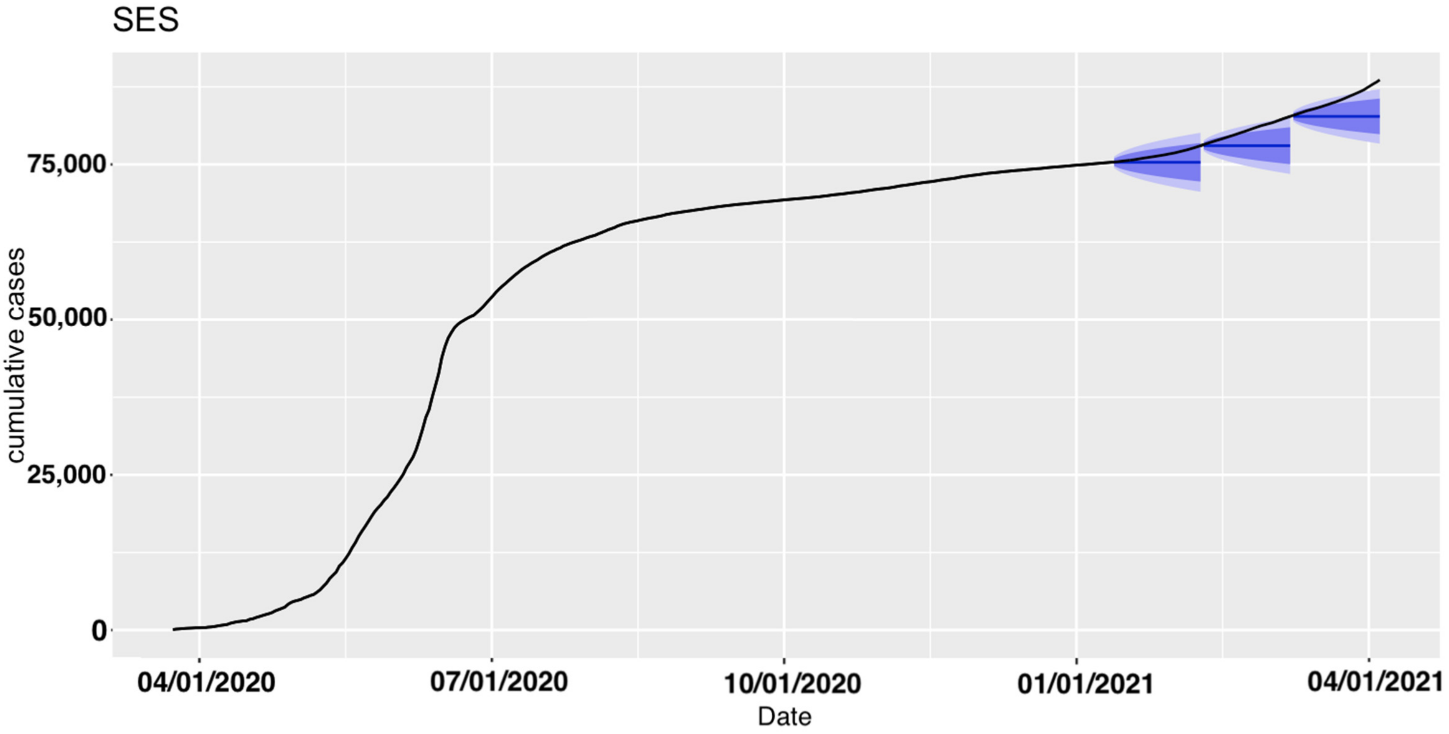

(f)HoltWinters

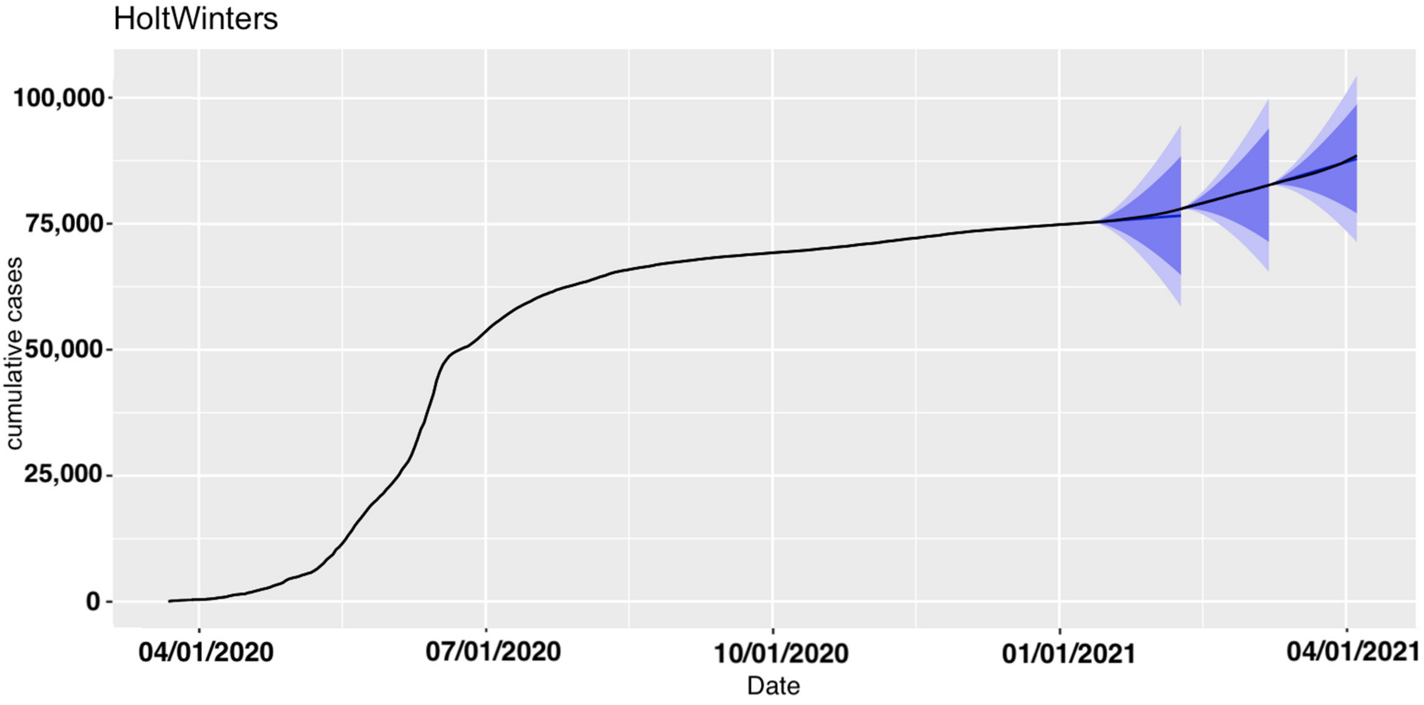

(g)cubic spline

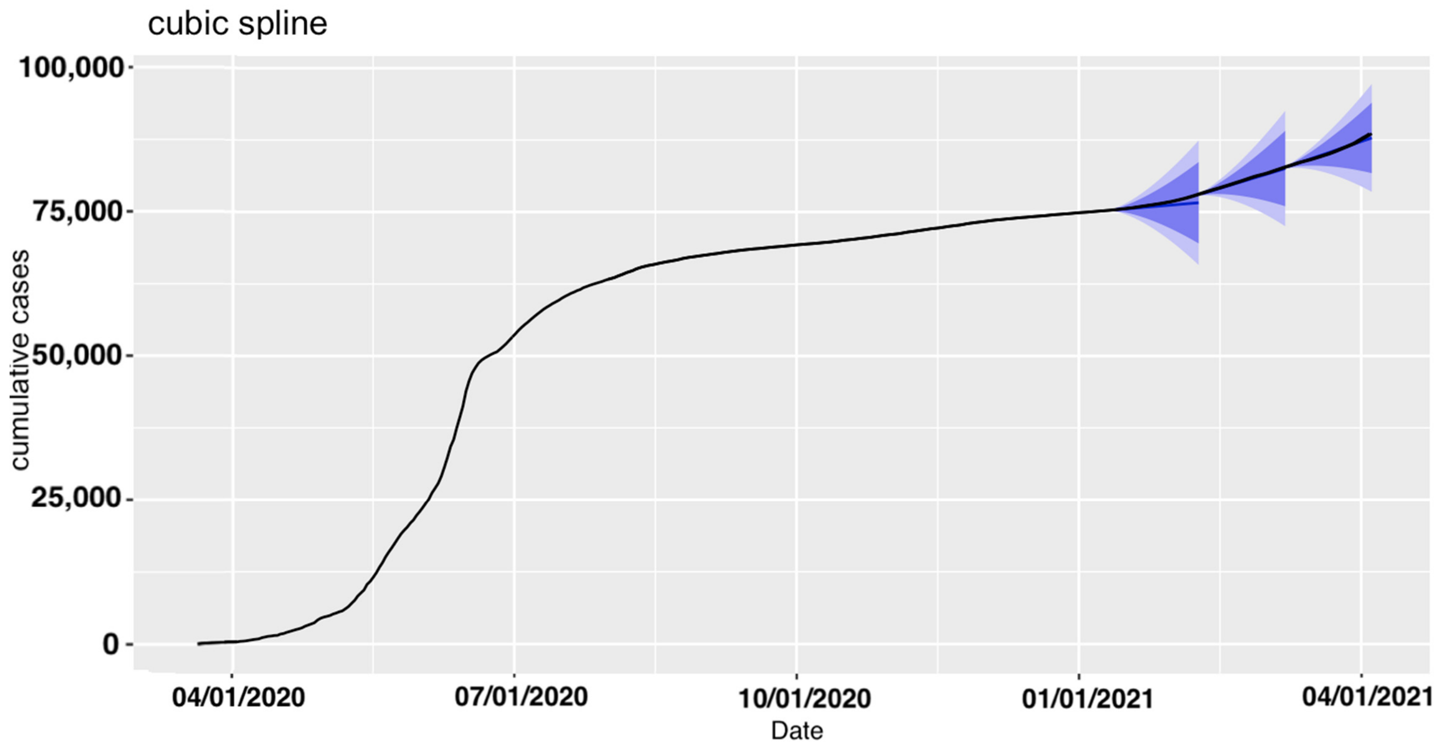

Supplement: Supplementary file 1 [file ijerph-18-08660-s001.zip › Supplementary File 2.pdf]
